# The serotonin transporter sustains human brown adipose tissue thermogenesis

**DOI:** 10.1038/s42255-023-00839-2

**Published:** 2023-08-03

**Authors:** Karla J. Suchacki, Lynne E. Ramage, T’ng Choong Kwok, Alexandra Kelman, Ben T. McNeill, Stewart Rodney, Matthew Keegan, Calum Gray, Gillian MacNaught, Dilip Patel, Alison M. Fletcher, Joanna P. Simpson, Roderick N. Carter, Robert K. Semple, Natalie Z. M. Homer, Nicholas M. Morton, Edwin J. R. van Beek, Sonia J. Wakelin, Roland H. Stimson

**Affiliations:** 1 University/BHF Centre for Cardiovascular Science, University of Edinburgh, The Queen’s Medical Research Institute, Edinburgh, UK; 2 Edinburgh Imaging Facility QMRI, University of Edinburgh, Edinburgh, UK; 3 Department of Medical Physics, Royal Infirmary of Edinburgh, Edinburgh, UK; 4 Mass Spectrometry Core, Edinburgh Clinical Research Facility, University/BHF Centre for Cardiovascular Science, University of Edinburgh, The Queen’s Medical Research Institute, Edinburgh, UK; 5 Department of Surgery, Royal Infirmary of Edinburgh, Edinburgh, UK

**Keywords:** Fat metabolism, Obesity, Transcriptomics

## Abstract

Activation of brown adipose tissue (BAT) in humans is a strategy to treat obesity and metabolic disease. Here we show that the serotonin transporter (SERT), encoded by *SLC6A4*, prevents serotonin-mediated suppression of human BAT function. RNA sequencing of human primary brown and white adipocytes shows that *SLC6A4* is highly expressed in human, but not murine, brown adipocytes and BAT. Serotonin decreases uncoupled respiration and reduces uncoupling protein 1 via the 5-HT_2B_ receptor. SERT inhibition by the selective serotonin reuptake inhibitor (SSRI) sertraline prevents uptake of extracellular serotonin, thereby potentiating serotonin’s suppressive effect on brown adipocytes. Furthermore, we see that sertraline reduces BAT activation in healthy volunteers, and SSRI-treated patients demonstrate no ^18^F-fluorodeoxyglucose uptake by BAT at room temperature, unlike matched controls. Inhibition of BAT thermogenesis may contribute to SSRI-induced weight gain and metabolic dysfunction, and reducing peripheral serotonin action may be an approach to treat obesity and metabolic disease.

## Abstract (web summary)

Suchacki et al. show that serotonin suppresses human brown adipose tissue (BAT) activation, and that inhibition of the serotonin transporter (SERT) potentiates the suppressive action of extracellular serotonin on BAT by preventing serotonin uptake.

## Main

The identification of BAT in adult humans approximately 15 years ago has rekindled interest in activating this tissue as a new strategy to treat obesity and associated metabolic diseases such as type 2 diabetes mellitus and dyslipidaemia^1^. BAT is a thermogenic organ that increases energy expenditure (EE) to generate heat in a process called non-shivering thermogenesis, maintaining body temperature in a cold environment^2^. This is achieved primarily through the unique thermogenic protein uncoupling protein 1 (UCP1), which allows transduction of the electron proton gradient to uncouple energy production from adenosine triphosphate synthesis in BAT. Human BAT retains many similarities to rodent BAT; for example, human BAT contributes to non-shivering thermogenesis^3^, contains functional UCP1 (ref. ^4^), is activated by cold^5,6^ and is under sympathetic regulation^7^. Human BAT demonstrates substantial glucose uptake, in addition to utilization of local triglyceride stores and other energy substrates, to fuel cold-induced thermogenesis (CIT)^8^. This high glucose uptake is exploited by the use of ^18^F-fluorodeoxyglucose-positron emission tomography (^18^F-FDG-PET), usually in combination with CT (PET–CT), to quantify BAT mass and act as a marker of activity in humans^9^. In the adult human, BAT is found in several locations such as the supraclavicular, axillary, paravertebral and peri-renal adipose depots, and BAT mass and activity are reduced in obesity^5,10,11^. In addition to increasing EE, BAT activation in humans improves insulin sensitivity^12^ and lipid clearance^13^, and the presence of BAT is associated with reduced cardiometabolic disease, particularly in obese individuals^14^. As such, there is substantial interest in activating BAT as a new treatment for cardiometabolic disease.

Initial proof of concept that BAT activity can be increased in adult humans came from studies using repeated intermittent cold exposure^15,16^. However, cold exposure is time-consuming and may cause discomfort, so pharmacotherapy to activate BAT at room temperature may be a more suitable therapeutic approach. To date, studies in adult humans have been mainly restricted to the sympathomimetic mirabegron (a β3-agonist), which increased EE and improved metabolic parameters; however, mirabegron increased blood pressure and heart rate which limits the potential for chronic administration^7,17,18^. Numerous factors have been identified that induce browning and increase EE in rodents such as fibroblast growth factor 21 and several bone morphogenic proteins^19^; however, none of these agents have yet led to successful therapeutics for patients. Importantly, there are differences in the regulation of human and murine BAT, as exemplified by glucocorticoids that acutely increase BAT activity in humans but decrease thermogenesis in rodents^20^. These results highlight the need to dissect BAT physiology in humans to understand the pathways regulating human BAT activation and develop safe therapeutics. Data from immortalized human brown adipocytes have provided some additional understanding of these pathways^21,22^; however, immortalization can alter the molecular signature of cells. Therefore, primary human brown adipocytes are an important model in which to identify new genes and pathways regulating human BAT function in vivo.

In this paper, we performed RNA sequencing (RNA-seq) of primary human brown and white adipocytes. Through this we identified the serotonergic pathway as a regulator of human BAT function. In particular, we identified high expression and an important role for the serotonin transporter (SERT) in human brown, but not white adipocytes; importantly SERT was not expressed in either murine brown or beige adipocytes. Furthermore, we have determined the importance of SERT both in vitro and in vivo, revealing that SERT plays a protective role to prevent serotonin-mediated suppression of human BAT activation.

## Results

### SERT is highly expressed in human brown adipocytes

**Fig. 1:**
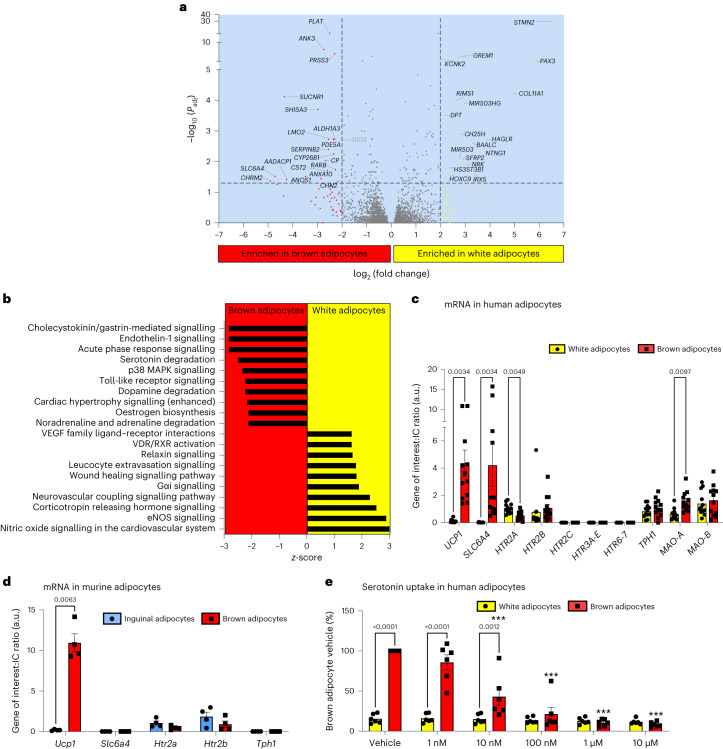
SERT is highly expressed in human but not murine brown adipocytes. **a**,**b**, Transcriptional profiling of paired human white and brown adipocytes (*n* = 4 per group; participant data detailed in Supplementary Information). **a**, Volcano plot of transcripts differentially expressed between brown and white adipocytes, with genes highlighted in black text representing those significantly DEGs (log_2_(fold change) >2 and adjusted *P* values (*P*_adj_) <0.05), DIO2 is highlighted in grey owing to the log_2_(fold change) being 1.99. **b**, Top canonical pathways enriched in paired human white and brown adipocytes with *z* scores >2. eNOS, endothelial nitric oxide synthase; RXR, retinoid X receptor; VDR, vitamin D receptor; VEGF, vascular endothelial growth factor. **c**,**d**, qPCR of paired human white (yellow columns) and brown adipocytes (red columns; *n* = 12 biologically independent samples per group) (**c**) and paired murine inguinal/beige (blue columns) and brown adipocytes (red columns; *n* = 4 biologically independent animals per group) (**d**). **e**, ^3^H-serotonin uptake with and without increasing concentrations of the SERT inhibitor sertraline (1 nM to 10 μM) in paired human white and brown adipocytes (*n* = 6 biologically independent samples per group). ***P < 0.0001 for sertraline concentrations ≥10 nM versus brown adipocyte vehicle. Data are mean ± s.e.m. Data were analysed using DESeq2 (**a**), Ingenuity Pathway Analysis (Qiagen) (**b**), multiple paired *t*-test (Holm–Sidak) (**c**,**d**) or two-way repeated measures ANOVA with Sidak multiple comparisons (**e**). Significant *P* values are detailed in the respective panels. IC, internal control. Source data

**Extended Data Fig. 1:**
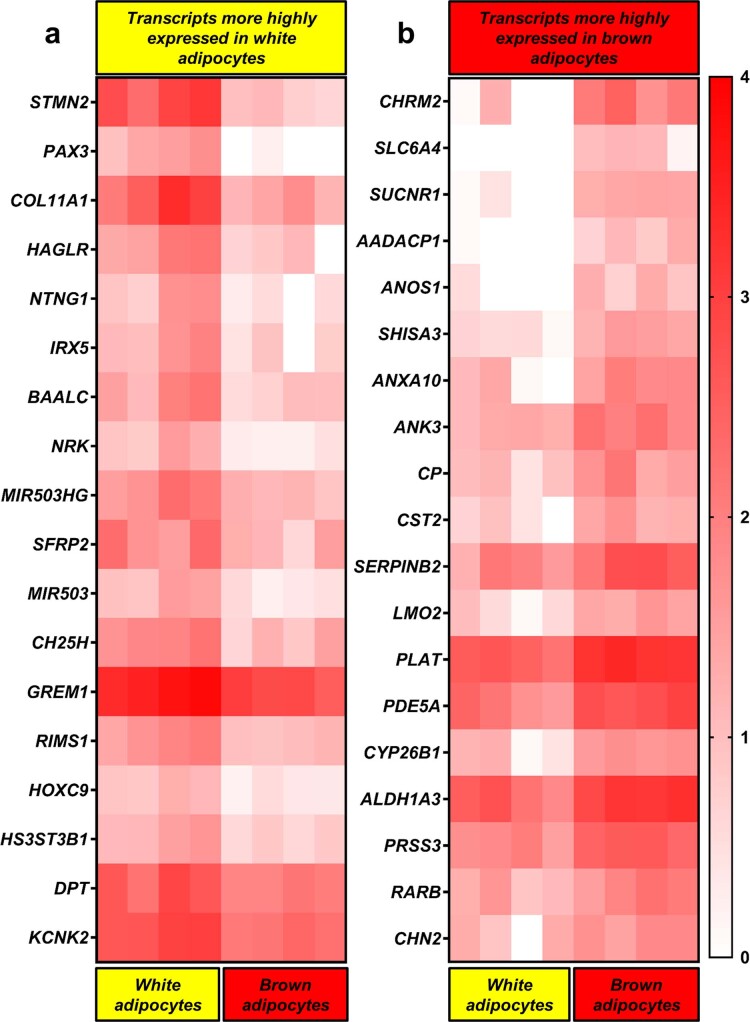
RNA sequencing of human primary brown and white adipocytes. Heatmaps of differentially expressed transcripts (those with > 2 log2-fold change and adjusted P value < 0.05) in paired human (**a**) white and (**b**) brown adipocytes. Transcripts are presented from top to bottom in decreasing order of differential expression between cell types, while the colour scale ranges from red (highest expression) to white (lowest expression). Log10 values ≤ 0 are presented as 0 on the heatmap. Source data

To identify new genes and pathways regulating human BAT function, we performed RNA-seq on paired human primary brown and white differentiated pre-adipocytes. We identified 185 transcripts with more than fourfold greater expression in human brown adipocytes and 235 transcripts with more than fourfold greater expression in white adipocytes (Fig. 1a and its Source data). In white adipocytes, *STMN2* and *PAX3* were the two most highly differentially expressed genes (DEGs) (Fig. 1a and Extended Data Fig. 1a), in line with published data^23^. As expected, genes such as *UCP1*, *DIO2*, *ADRB1* and *PRDM16* were enriched in human brown adipocytes (see Source data for Fig. 1a and https://www.ebi.ac.uk/biostudies/arrayexpress/studies/E-MTAB-10123), but none of the top DEGs were those typically associated with BAT function (Fig. 1a and Extended Data Fig. 1b). The two top DEGs were *SLC6A4* (encoding SERT) and *CHRM2* (which encodes the type 2 muscarinic acetylcholine receptor), both were expressed >25-fold more highly in human brown compared with white adipocytes (Fig. 1a and Extended Data Fig. 1b). We performed pathway analysis to identify canonical pathways whose components were more highly expressed in brown adipocytes (Fig. 1b). Of most interest was the serotonin degradation pathway, highlighting a possible role for serotonin metabolism in BAT function (Fig. 1b). This led us to prioritize and investigate the role of serotonin uptake by *SLC6A4* in human BAT.

Serotonin (5-hydroxytryptamine or 5-HT) is a neurotransmitter involved in multiple processes including the regulation of energy balance. For example, serotonin acts centrally to reduce appetite through the 5-HT_1B_ and 5-HT_2C_ receptors^24–26^. There is also growing evidence that peripheral serotonin action has deleterious effects on metabolic health and BAT function^27^. Serotonin does not cross the blood–brain barrier, consequently central and peripheral serotonin levels are under separate control. The type 1 isoform of tryptophan hydroxylase (TPH1) is the rate-limiting enzyme in peripheral serotonin synthesis. Mice lacking *Tph1* globally or selectively in adipose tissue have reduced weight gain, increased BAT thermogenesis and improved glucose tolerance and insulin sensitivity on a high-fat diet^28,29^. Therefore, we hypothesized that serotonin uptake by SERT regulates brown adipocyte function by reducing extracellular serotonin concentrations and resultant activation of 5-HT receptors. We first performed quantitative polymerase chain reaction analysis (qPCR) to validate the RNA-seq data; this confirmed that *SLC6A4* messenger RNA levels were >200-fold greater in human brown compared with white adipocytes (Fig. 1c). The serotonin-metabolizing enzyme monoamine oxidase A (*MAO-A*) was also more highly expressed in brown adipocytes (Fig. 1c). Both the serotonin 2A and 2B receptors (5-HT_2A/2B_; *HTR2A/B*) were expressed in human brown and white adipocytes, although 5-HT_2A_ mRNA levels were lower in brown adipocytes (Fig. 1c). The 5-HT_2C_ (*HTR2C*), 5-HT_3A-E_ (*HTR3A-E*), 5-HT_6_ and 5-HT_7_ receptors were not expressed in either cell type; the lack of 5-HT_3A_ expression is of note because this receptor has been implicated in serotonin-mediated suppression of murine BAT thermogenesis^28^. There was no substantial expression of other 5-HT receptor subtypes in human brown adipocytes in the RNA-seq data (Extended Data Table 1).

To determine whether expression patterns were similar between species, we performed qPCR analysis in murine primary brown and inguinal (beige) adipocytes. Although the 5-HT_2A/2B_ receptors were expressed in brown and inguinal adipocytes, *Slc6a4* was not detectable in either cell type (Fig. 1d), highlighting a major species-specific difference in transporter expression. To confirm the role of SERT, we incubated primary human brown and white adipocytes in medium containing ^3^H-serotonin with and without increasing concentrations of the SERT inhibitor sertraline (a selective serotonin reuptake inhibitor or SSRI), and measured ^3^H-serotonin uptake in cell lysates. ^3^H-Serotonin uptake was substantially higher in brown adipocytes and was dose-dependently inhibited by sertraline (Fig. 1e). These data demonstrate the presence of functional SERT protein in brown, but not white adipocytes, and confirm that SERT is responsible for serotonin uptake by human brown adipocytes.

### SERT inhibition potentiates serotonin-mediated suppression

**Fig. 2:**
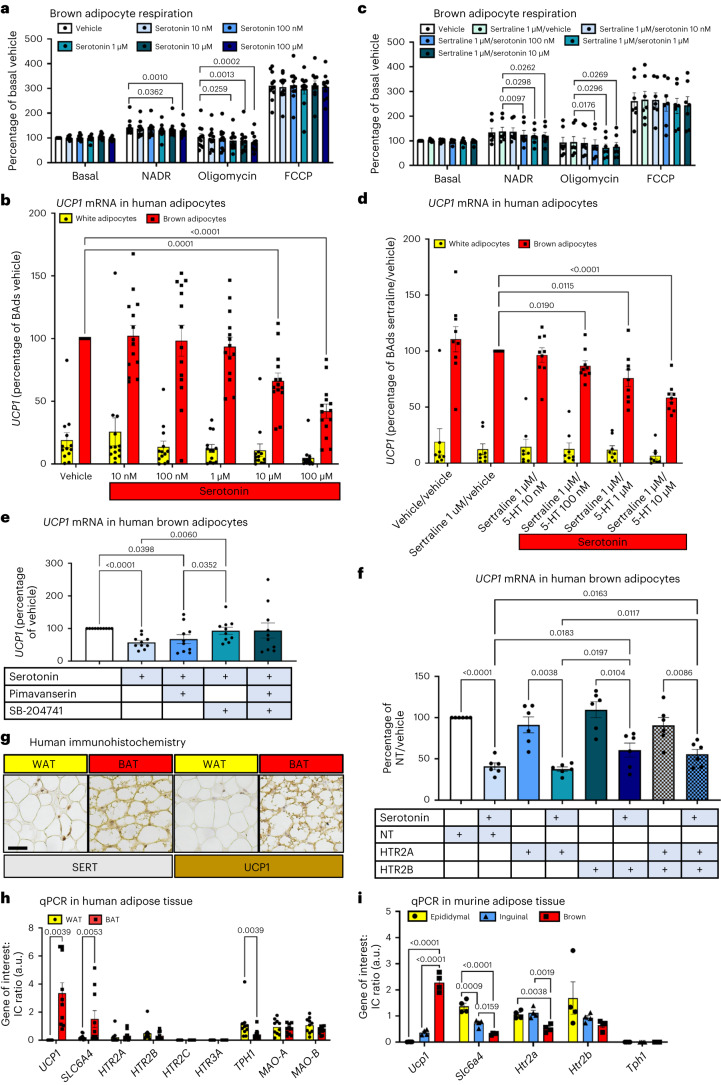
Serotonin inhibits UCP1 through the 5-HT_2B_ receptor in human brown adipocytes. **a**, OCR in human brown adipocytes (*n* = 11 biologically independent samples) cultured in vehicle or serotonin (10 nM to 100 μM) for 24 h, presented as the percentage of basal OCR during vehicle. NADR, noradrenaline. **b**, *UCP1* mRNA levels in paired human white (yellow columns; *n* = 13 biologically independent samples) and brown (red; *n* = 14 biologically independent samples) adipocytes incubated with vehicle or ascending serotonin concentrations. BAds, brown adipocytes. **c**, OCR in human brown adipocytes (*n* = 7 biologically independent samples) cultured in vehicle, sertraline (1 μM) or sertraline plus serotonin (10 nM to 10 μM) for 24 h, presented as the percentage of basal OCR during vehicle. **d**, *UCP1* mRNA levels in paired human white (*n* = 8 biologically independent samples) and brown (*n* = 9 biologically independent samples) adipocytes incubated with vehicle, sertraline (1 μM) or sertraline plus serotonin (10 nM to 10 μM) for 24 h. **e**, *UCP1* mRNA levels in primary human brown adipocytes incubated with 100 μM serotonin with or without the inverse 5-HT_2A_ agonist pimavanserin (10 μM) or the 5-HT_2B_ receptor antagonist SB-204741 (10 μM) (*n* = 10 biologically independent samples). **f**, *UCP1* mRNA levels in primary human brown adipocytes (*n* = 6 biologically independent samples) incubated with either vehicle or 100 μM serotonin for 24 h following siRNA-knockdown of HTR2A, HTR2B, both or an NT control. Significant *P* values are shown for comparisons between the respective vehicles of each group and between the groups of serotonin-treated cells. **g**, Immunohistochemistry of human WAT (yellow) and BAT (red), expression of SERT (left) and UCP1 (right) in BAT indicated by brown staining. Scale bar, 40 μm. **h**, mRNA expression in human BAT versus WAT (*n* = 10 biologically independent samples). **i**, mRNA expression in murine BAT (red) versus inguinal (beige; blue) or epididymal WAT (yellow) (*n* = 4 biologically independent samples). Data are mean ± s.e.m. Data were analysed by one-way (**a**,**c**,**e**,**f**) or two-way (**b**,**d**) repeated measures ANOVA, Wilcoxon signed-rank test (**h**) or one-way ANOVA (**i**). Significant *P* values are detailed in the respective panels. Source data

**Extended Data Fig. 2:**
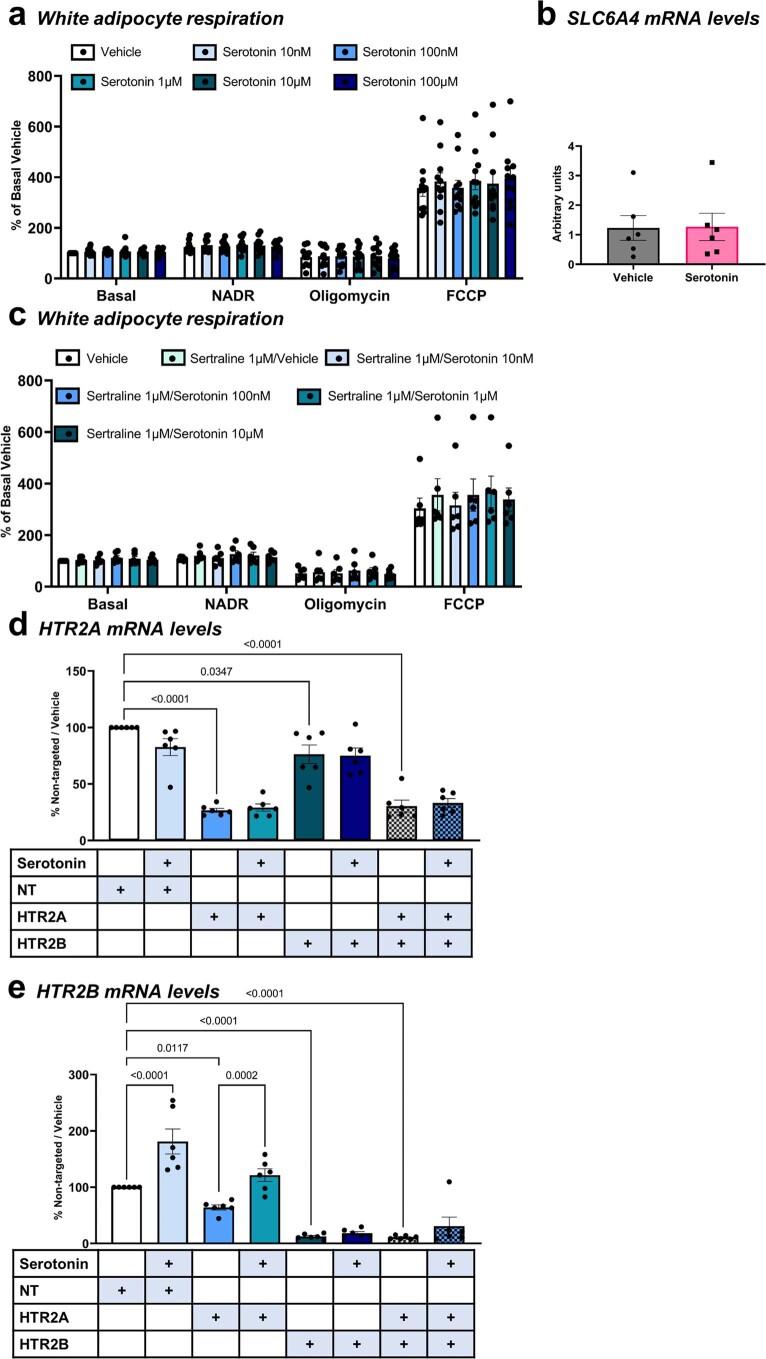
Serotonin alters uncoupled respiration in human brown but not white adipocytes. (**a**) Oxygen consumption rates (OCR) in human white adipocytes (n = 11 biologically independent samples) cultured in vehicle or serotonin (10nM-100μM) for 24 hours prior to quantifying OCR during basal conditions and following sequential addition of noradrenaline, oligomycin and carbonyl cyanide-p-trifluoromethoxyphenylhydrazone (FCCP). Data are presented as the % of basal OCR during vehicle. (**b**) mRNA levels of *SLC6A4* in human brown adipocytes (n = 6 biologically independent samples) that were cultured in vehicle or 100 µM serotonin. (**c**) Oxygen consumption rates (OCR) in human white adipocytes (n = 6 biologically independent samples) cultured in vehicle, sertraline (1 μM), or sertraline plus serotonin (10nM-10μM) for 24 hours, presented as the % of basal OCR during vehicle. (**d**-**e**) mRNA levels of (**d**) *HTR2A* and (**e**) *HTR2B* in primary human brown adipocytes (n = 6 biologically independent samples) incubated with either vehicle or 100 μM serotonin for 24 hours following siRNA-knockdown of *HTR2A*, *HTR2B*, both receptors or a non-targeted (NT) control. Data are mean ± SEM and were analysed by (a, c-e) one-way repeated measures ANOVA or (b) paired t-test. For d-e, significant P values are shown for the effect of knockdown in each vehicle treated-group and for the effect of serotonin compared to their respective vehicle. Source data

Serotonin has a direct suppressive effect on BAT thermogenesis in rodents; for example, in vitro serotonin decreased oxygen consumption by murine brown adipocytes by decreasing sensitivity to adrenergic stimulation^29^, an effect possibly mediated via the 5-HT_3A_ receptor^28^. In vivo, mice with global disruption of *Tph1* or given TPH1 inhibitors have improved BAT function^29^. Tissue-specific disruption of *Tph1* either in adipose tissue or in mast cells also improves brown and beige adipose thermogenic function^28,30^, suggesting a local reduction in serotonin levels improves BAT function. Therefore, we tested whether serotonin regulates human brown and white adipocyte respiration. Paired human brown and white adipocytes were cultured in ascending concentrations of serotonin for 24 h before analysis. Serotonin concentrations of 1 μM and above inhibited noradrenaline-stimulated uncoupled respiration in human brown but not white adipocytes (Fig. 2a and Extended Data Fig. 2a), revealing that serotonin directly inhibits human brown adipocyte function. To determine whether serotonin suppresses brown adipocyte function through the key thermogenic protein UCP1, we cultured brown and white adipocytes in ascending concentrations of serotonin for 24 h and performed qPCR analysis. Both 10 μM and 100 μM serotonin reduced *UCP1* mRNA levels in brown and white adipocytes (Fig. 2b), whereas 100 μM serotonin did not alter *SLC6A4* mRNA levels in human brown adipocytes (Extended Data Fig. 2b).

To determine the importance of SERT, primary brown and white adipocytes were cultured in the presence of 1 μM sertraline with ascending concentrations of serotonin for 24 h. Sertraline had no effect on brown adipocyte respiration in the absence of serotonin, but serotonin concentrations of 100 nM and above inhibited uncoupled respiration in human brown but not white adipocytes (Fig. 2c and Extended Data Fig. 2c). Similarly, serotonin concentrations of 100 nM and above inhibited *UCP1* mRNA levels in the presence of sertraline in human brown adipocytes (Fig. 2d). These data demonstrate that SERT inhibition augments serotonin’s suppressive effect on human brown adipocyte function through preventing uptake of extracellular serotonin.

### Serotonin inhibits UCP1 via the 5-HT_2B_ receptor

Next, to determine whether serotonin suppresses UCP1 through the 5-HT_2A_ and/or 5-HT_2B_ receptors, the only 5-HT receptors we found to be expressed in human brown adipocytes, we incubated primary human brown adipocytes with 100 μM serotonin with or without the selective 5-HT_2A_ receptor inverse agonist pimavanserin or the 5-HT_2B_ receptor antagonist SB-204741 for 24 h. SB-204741, but not pimavanserin, prevented serotonin from inhibiting *UCP1* (Fig. 2e), indicating that serotonin suppresses UCP1 through the 5-HT_2B_ receptor. To confirm this, we repeated this experiment but this time used small interfering RNA-mediated knockdown of *HTR2A* and/or *HTR2B*. *HTR2A* and *HTR2B* mRNA levels were reduced 70–90% by knockdown compared with non-targeted (NT) controls (Extended Data Fig. 2d,e). *HTR2A* and *HTR2B* knockdown also reduced mRNA levels of the other receptor compared with NT control, albeit to a far lesser extent (Extended Data Fig. 2d,e). Similar to previous experiments, serotonin decreased *UCP1* mRNA levels by ~60% in wells treated with NT controls and also in those with *HTR2A* knockdown (Fig. 2f). Serotonin suppressed *UCP1* levels to a lesser extent in brown adipocytes with knockdown of *HTR2B*, consistent with the 5-HT_2B_ receptor mediating at least part of this effect (Fig. 2f). In addition, serotonin substantially increased *HTR2B* but not *HTR2A* mRNA levels, in keeping with a regulatory mechanism potentiating serotonin-mediated suppression of brown adipocyte thermogenesis (Extended Data Fig. 2e,f). The partial reversal of serotonin’s effect on UCP1 is most probably due to incomplete knockdown of the 5-HT_2B_ receptor, particularly as SB-204741 fully prevented this effect and no other serotonin receptors were expressed in these cells.

To determine whether SERT was important in vivo, we first performed immunohistochemistry and qPCR analysis in paired human BAT and white adipose tissue (WAT). SERT protein (Fig. 2g) and mRNA levels (Fig. 2h) were increased substantially in BAT versus WAT. *TPH1* mRNA levels were also reduced in BAT, in keeping with reduced local peripheral serotonin synthesis. Similar to the in vitro data, both 5-HT_2A_ and 5-HT_2B_ receptors were expressed in BAT and WAT, but 5-HT_2C_ or 5-HT_3A_ receptors were not (Fig. 2h). We then measured *Slc6a4* mRNA levels in BAT, inguinal (beige) WAT and epididymal WAT in 8–9-week-old 129/Ola male mice. *Slc6a4* expression was highest in epididymal WAT, intermediate in inguinal WAT and lowest in BAT, in opposition to the expression patterns of *Ucp1* (Fig. 2i). 5-HT_2A_ receptor expression was lower in murine BAT, whereas 5-HT_2B_ receptors were similar across all three depots. These data reveal that the species-specific differences in SERT expression are retained in vivo, therefore SERT could potentially provide humans with greater protection from the suppressive effects of serotonin on BAT thermogenesis compared with rodents.

### SERT inhibition reduces BAT glucose uptake in vivo in humans

**Fig. 3:**
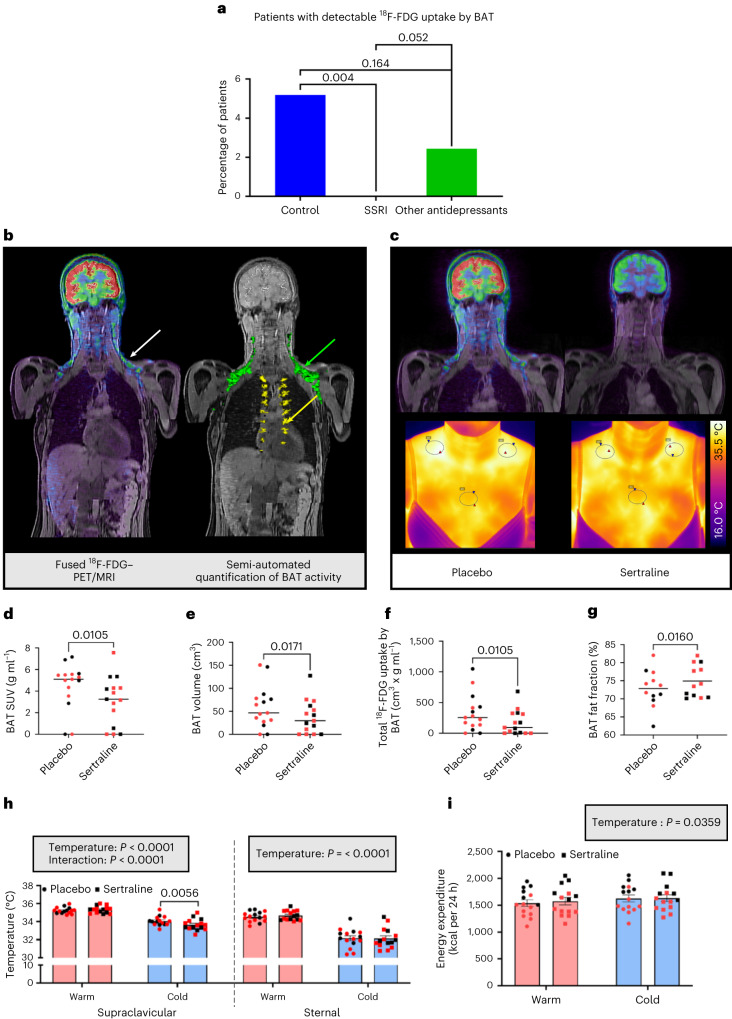
SERT inhibition suppresses BAT thermogenesis in vivo in humans. **a**, Retrospective analysis of ^18^F-FDG-PET–CT scans performed at room temperature in patients treated with SSRIs (*n* = 153), those prescribed other classes of antidepressants (*n* = 164) and matched controls (*n* = 270). **b**, Left: fused PET–MRI (^18^F-FDG uptake by supraclavicular BAT (white arrow)). Right: three-dimensional PET–MRI with quantification of ^18^F-FDG uptake by cervical/supraclavicular/axillary (green) and paraspinal (yellow) BAT. **c**, Coronal fused PET–MRI (upper) and thermal images (lower) of a representative participant on placebo (left) and sertraline (right) phases. In the thermal images, regions of interest (ovals) are drawn around the left and right supraclavicular (to highlight areas of BAT) and sternal (control) areas. **d**–**g**, Quantification of ^18^F-FDG uptake by BAT (**d**), BAT volume (**e**), total ^18^F-FDG uptake by BAT (**f**) and BAT fat fraction (**g**) on placebo (circles) and sertraline (squares) phases in male (black) and female (red) participants (*n* = 15 (**d**–**f**) or *n* = 12 (**g**) with detectable ^18^F-FDG uptake on both phases). Lines depict median (**d**–**f**) and mean (**g**) values. **h**, Supraclavicular and sternal skin temperatures during warm exposure (pink columns) and cold exposure (blue columns) (*n* = 15 biologically independent participants). **i**, Whole-body EE measured by indirect calorimetry (*n* = 15 biologically independent participants). EE was higher in males (*P* < 0.001). Data are median (**d**–**f**) or mean ± s.e.m. (**g**–**i**). Data were analysed by chi-squared test (**a**), Wilcoxon signed-rank test (**d**–**f**), paired *t*-test (**g**) or two-way repeated measures ANOVA (**h**,**i**). Significant *P* values are detailed in the respective panels. Source data

We hypothesized that SERT inhibition would increase the suppressive effect of serotonin on BAT function. SERT inhibitors known as SSRIs are the most commonly prescribed class of pharmacotherapy used to treat depression^31^. Therefore, we analysed approximately 600 ^18^F-FDG-PET–CT scans that had been performed in patients as part of their clinical care at standard room temperature (approximately 20–21 °C). PET–CT scans were analysed from three matched groups: (1) control patients not prescribed any antidepressant medication; (2) patients prescribed SSRIs; and (3) patients prescribed other classes of antidepressant medication (Extended Data Table 2). None of the SSRI-treated patients had detectable ^18^F-FDG uptake by BAT, compared with ~5% of controls (similar to published data at room temperature^20,32^) and ~2.5% of patients prescribed other antidepressant medications (*P* = 0.004 between SSRI and control groups; *P* = 0.052 between SSRI and other antidepressant groups) (Fig. 3a). In line with previous data, individuals with detectable ^18^F-FDG uptake by BAT at room temperature were more likely to be female and have a lower body mass index (BMI) than those without detectable BAT glucose uptake (Extended Data Table 3)^32,33^.

**Extended Data Fig. 3:**
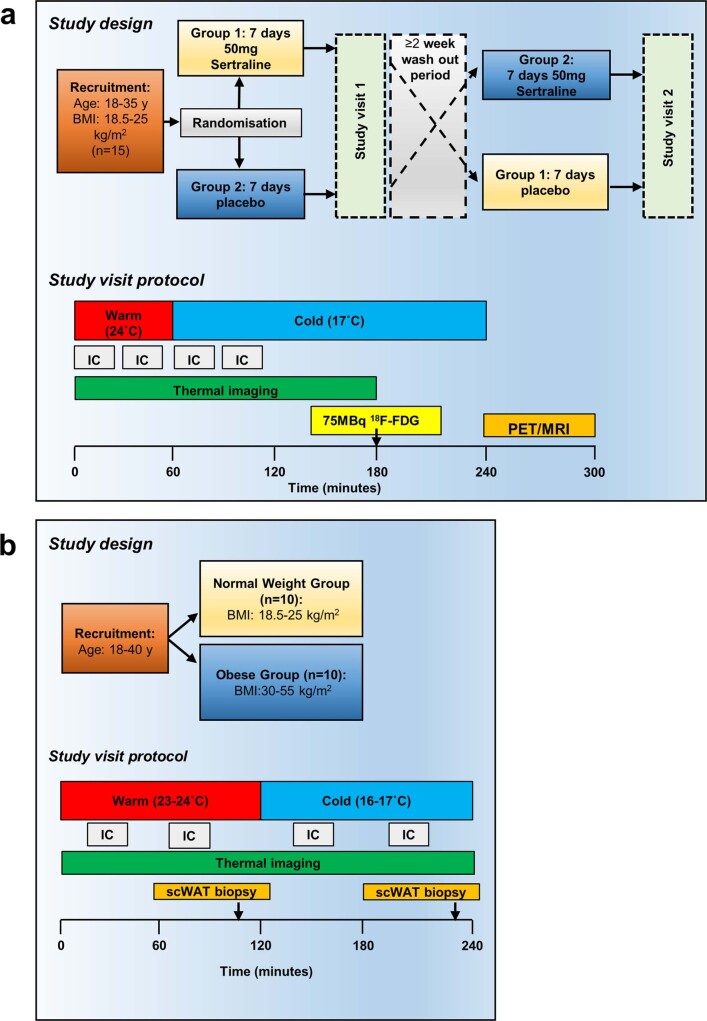
*In vivo* study protocols. (**a**) Fifteen participants (nine female, six male) aged 18-35 years with a normal BMI (18.5-25 kg/m^2^) were recruited to a randomised, double-blind, placebo-controlled crossover study to determine the effect of the SSRI sertraline (50 mg for 7 days) on BAT activity. At each study visit, BAT activity was quantified using ^18^F-fluorodeoxyglucose (^18^F-FDG) PET/MRI and thermal imaging while whole body energy expenditure was measured using indirect calorimetry (IC). Participants spent 1 hour in a warm room, then 3 hours in a cold room to activate BAT. After 2 hours of cold exposure, 75 MBq of ^18^F-FDG was injected intravenously and a PET/MRI scan performed 1 hour later. (**b**) To determine the effect of obesity on circulating and adipose serotonin concentrations during cold exposure, male and female participants aged 18-40 years who were either normal weight (BMI 18.5-25 kg/m^2^) or obese (BMI 30-55 kg/m^2^) were recruited to a case-control study (n = 10/ group). Participants lay supine in a warm room for 2 hours prior to transfer to a cold room for 2 hours to activate BAT. BAT activity was measured by thermal imaging and whole body energy expenditure was measured by IC. Subcutaneous abdominal white adipose tissue (scWAT) biopsies were obtained following 105 minutes in each room. Source data

**Extended Data Fig. 4:**
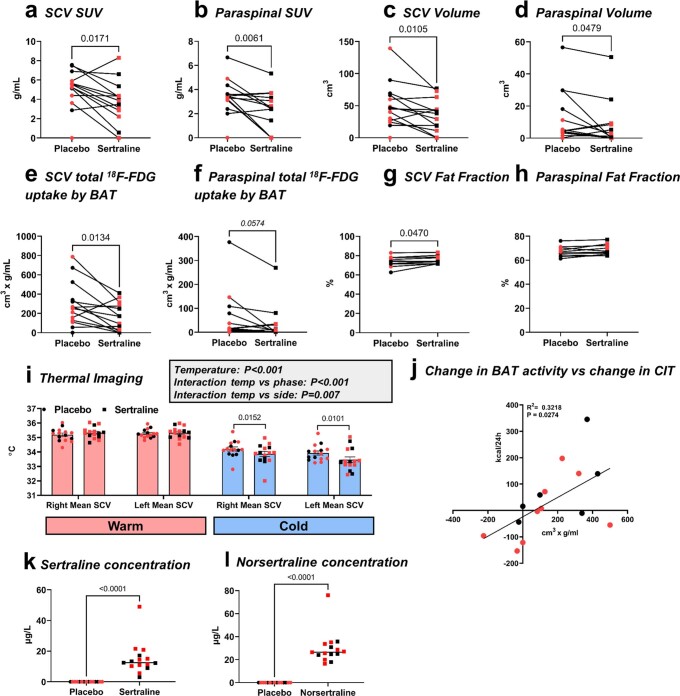
Sertraline inhibits BAT activity in supraclavicular and paraspinal depots. (**a-h**) ^18^F-FDG uptake by BAT was categorised into either supraclavicular/ cervical/ axillary (grouped as SCV) or paraspinal regions of interest on placebo (closed circles) and sertraline (closed squares) phases (a-f n = 15 and g-h n = 12). (**i**) Left and right supraclavicular (SCV) skin temperatures during warm (pink columns) and cold exposure (blue columns, both n = 15). Left SCV temperature was lower than right SCV during cold exposure but the effect of sertraline was similar in both SCV regions. (**j**) The correlation in the change in total ^18^F-FDG uptake by BAT (BAT activity) between placebo and sertraline phases with the change in cold-induced thermogenesis (CIT) between phases (n = 15). (**k-i**) Circulating (**k**) sertraline and (**l**) norsertraline drug concentrations (n = 15), lines detail the median values. Male and female subjects are depicted by black and red circles / squares respectively. Data are mean ± SEM and were analysed using (a-f, k-l) Wilcoxon signed-rank test, (g-h) paired t test, (i) two-way repeated measures ANOVA or (j) Pearson correlation. Significant P values are detailed in the respective panels. Source data

These data suggested that SSRIs inhibit BAT activation. To confirm this we recruited 15 healthy participants to a randomized double-blind crossover study (Extended Data Fig. 3a and Table 1). Volunteers were given the SSRI sertraline (50 mg per day) or placebo for 7 days before undergoing ^18^F-FDG-PET–magnetic resonance imaging (MRI) and thermal imaging (Fig. 3b,c) during mild cold exposure (approximately 16–17 °C). Detectable ^18^F-FDG uptake by BAT during cold exposure (Fig. 3b,c) was observed on at least one phase in 13 participants (eight of the nine females and five of the six males). Sertraline substantially decreased ^18^F-FDG uptake by BAT and detectable BAT volume in all BAT depots (Fig. 3c–f and Extended Data Fig. 4a–f), and increased BAT fat fraction during cold exposure (Fig. 3g and Extended Data Fig. 4g,h). In addition, sertraline did not alter supraclavicular temperature during warm exposure, but decreased supraclavicular temperature (and similarly in left and supraclavicular right regions) without altering sternal skin temperature during cold exposure (Fig. 3c,h and Extended Data Fig. 4i). Sertraline had a similar effect in both sexes (Fig. 3d–h and Extended Data Fig. 4a–i). Cold exposure increased whole-body EE (also known as CIT, measured using indirect calorimetry) similarly on both phases (88 ± 33 kcal per 24 h on placebo versus 55 ± 39 kcal per 24 h on sertraline, respectively; *P* > 0.3) (Fig. 3i). The difference in ^18^F-FDG uptake by BAT between the SSRI and placebo phases correlated with the difference in CIT (Extended Data Fig. 4j). Circulating sertraline and its active metabolite norsertraline (Extended Data Fig. 4k,l) were detected only on the sertraline phase, but concentrations did not correlate with any measurements of BAT activity (see Source data for Fig. 3 and Extended Data Fig. 4).

**Table 1:** Participant data from randomized crossover study of sertraline

	Placebo	Sertraline	*P* value
Sex	9 females, 6 males		
Age (years)	24.7 ± 1.0	24.6 ± 1.0	0.568
Weight (kg)	65.0 ± 2.4	64.1 ± 2.2	**0.029**
Height (m)	1.70 ± 0.02	1.70 ± 0.02	0.065
BMI (kg m^−^^2^)	22.4 ± 0.5	22.0 ± 0.4	**0.005**
Fat mass (kg)	13.9 ± 1.4	11.9 ± 1.1	**0.008**
Fat mass (%)	19.9 ± 1.7	20.0 ± 2.3	0.960
Systolic blood pressure (mmHg)	127.1 ± 4.3	129.7 ± 5.0	0.492
Diastolic blood pressure (mmHg)	76.9 ± 3.0	76.1 ± 3.4	0.814
Outdoor temperature (°C)	6.3 ± 1.0	8.1 ± 1.2	0.266
Warm room temperature (°C)	23.3 ± 0.2	23.6 ± 0.2	0.294
Cold room temperature (°C)	16.7 ± 0.1	16.6 ± 0.1	0.267

Data are mean ± s.e.m. for all 15 participants. Outdoor temperature was measured at 10 a.m. on the morning of each study visit. The outdoor, warm and cold room temperatures were similar on both sertraline and placebo phases. Data were analysed using the paired *t*-test and significant *P* values are given in bold.

Source data

**Fig. 4:**
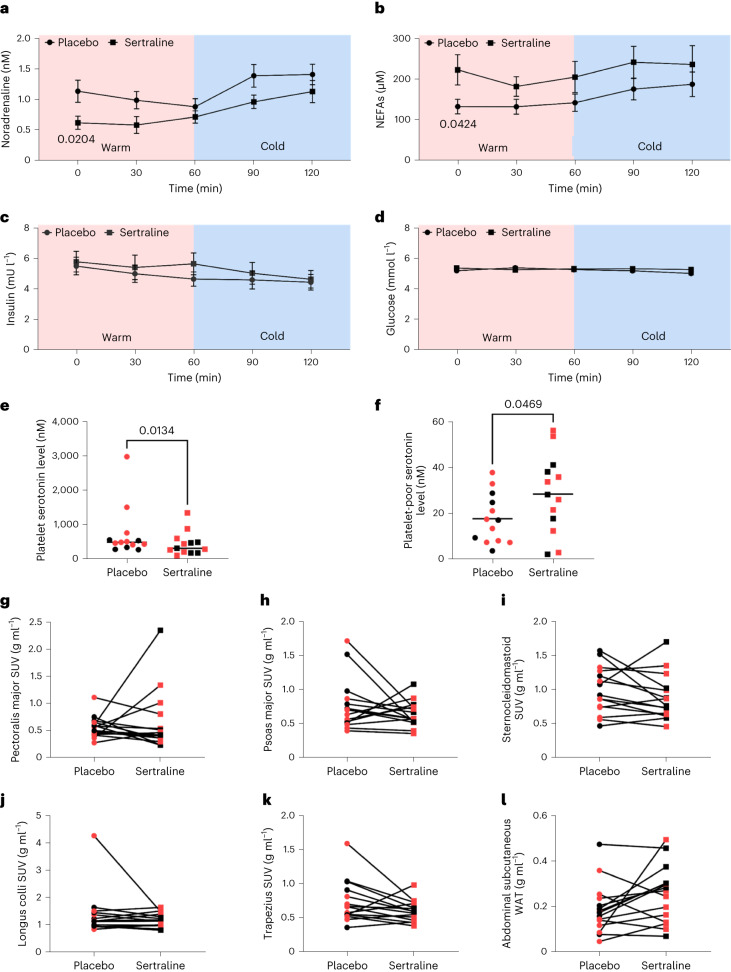
Sertraline does not alter hormonal responses to cold or ^18^F-FDG uptake by skeletal muscle. **a**–**d**, Circulating concentrations of noradrenaline (**a**), NEFAs (**b**), insulin (**c**) and glucose (**d**) (all *n* = 15 biologically independent participants per group) on sertraline (black squares) and placebo (black circles) phases (Extended Data Fig. 3a). **e**,**f**, Serotonin concentrations at baseline in platelets (**e**) and platelet-poor plasma (**f**) (both *n* = 13 per group). Lines depict the median (**e**) and mean (**f**) values. **g**–**l**, ^18^F-FDG uptake during cold exposure was quantified in pectoralis major (**g**), psoas major (**h**), sternocleidomastoid (**i**), longus colli (**j**) and trapezius skeletal (**k**) muscles, in addition to abdominal subcutaneous WAT (**l**) (all *n* = 15 per group) on both placebo (circles) and sertraline (squares) phases. Male and female participants are depicted by black and red circles/squares, respectively. Data are mean ± s.e.m. and were analysed by two-way repeated measures ANOVA (**a**–**d**), Wilcoxon signed-rank test (**e**) and paired *t*-test (**f**–**l**). Significant *P* values are detailed in the respective panels. Source data

Sertraline acutely reduced body weight by ~1 kg (Table 1), consistent with previous data^34^. Cold exposure increased circulating noradrenaline and non-esterified fatty acid (NEFA) concentrations (Fig. 4a,b)^35^. Sertraline reduced basal noradrenaline and increased NEFA concentrations compared with placebo; however, cold exposure induced a similar increase during both phases. Sertraline did not alter insulin or glucose concentrations (Fig. 4c,d), although cold exposure/time reduced serum insulin. SERT is vital to transport gut-derived serotonin to the circulation, but also for transport from the circulation to platelets where the majority of peripheral serotonin is stored^36,37^; as such, only free serotonin is biologically active, which mediates its effects via activation of the serotonin receptors. To determine the effect of sertraline on basal platelet and circulating serotonin levels, serotonin was measured in the platelet and platelet-poor (an estimate of free serotonin) fractions on both phases. Sertraline substantially decreased platelet serotonin concentrations but increased serotonin levels in platelet-poor plasma, consistent with increased free biologically active serotonin levels on the SSRI phase (Fig. 4e,f). Importantly, sertraline did not alter ^18^F-FDG uptake by skeletal muscles that contribute to CIT^38^ or by abdominal subcutaneous WAT during cold exposure (Fig. 4g–l), in keeping with a selective effect in BAT.

### Cold exposure acutely decreases circulating serotonin levels

**Fig. 5:**
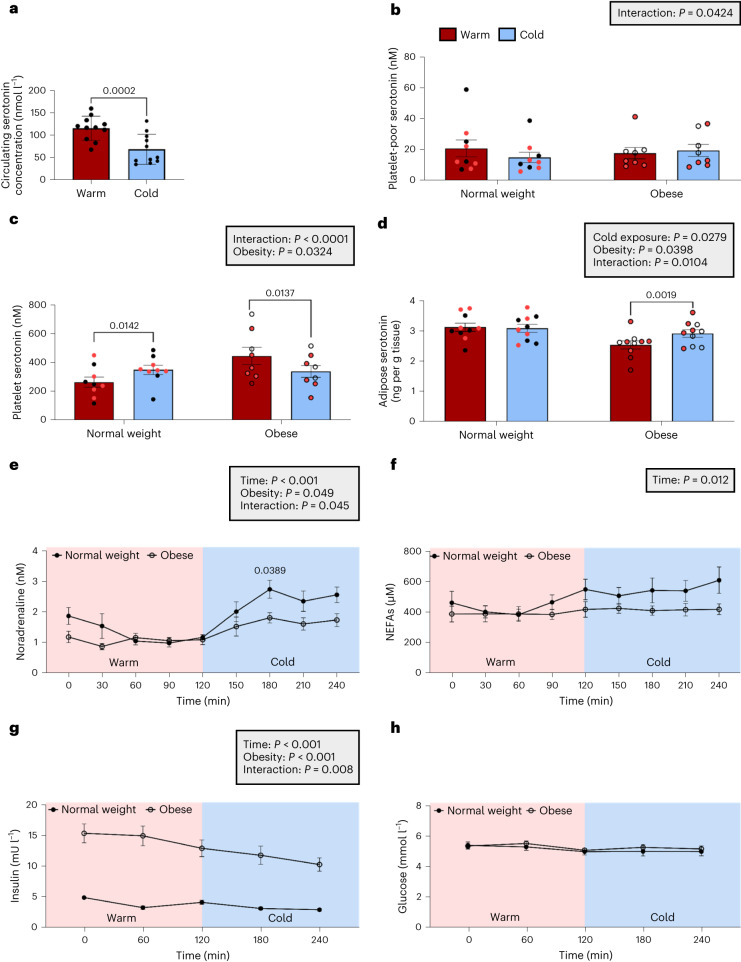
Obesity alters circulating and adipose serotonin concentrations during cold exposure in humans. **a**, Circulating plasma serotonin concentrations during warm conditions (red columns) and mild cold exposure (16–17 °C; blue columns) in normal weight BAT-positive men (*n* = 11). **b**–**d**, Serotonin levels were measured in platelet-poor plasma (**b**) (*n* = 17), platelets (**c**) (*n* = 17) and abdominal WAT (**d**) (*n* = 20) during warm (red columns) and cold (blue columns) conditions in normal weight (closed circles) and obese (open circles) participants. Male and female participants are depicted by black and red circles, respectively. **e**–**h**, Circulating hormones/intermediates were measured in normal weight and obese participants (*n* = 10 biologically independent participants per group) during warm and cold exposure: circulating noradrenaline (**e**), NEFAs (**f**), insulin (**g**) and glucose (**h**). Data are mean ± s.e.m. and were analysed by paired *t*-test (**a**) and two-way-repeated measures ANOVA (**b**–**h**). Insulin (**g**) was greater in obese participants (*P* < 0.0001 at all time points); otherwise, significant *P* values are detailed in the respective panels. Source data

To determine whether cold exposure alters circulating serotonin concentrations, we analysed plasma samples from 11 previously reported BAT-positive participants with normal BMI^20,35^. Acute mild cold exposure at 16–17 °C for less than 2 h reduced circulating serotonin concentrations by ~40% (Fig. 5a). We hypothesized that this cold-induced reduction in circulating serotonin may be due to increased platelet serotonin uptake. Because serotonin suppresses brown adipocyte thermogenesis (Fig. 2), reduction of circulating serotonin concentrations during cold exposure could enhance BAT thermogenesis, assuming this also reduces extracellular serotonin concentrations in BAT. Obesity may be associated with reduced BAT activity^5,11^ and increased circulating serotonin concentrations^39^, so we tested whether circulating (platelet and platelet-poor) and abdominal WAT serotonin concentrations were altered in obesity during warm and cold exposure. Thermal imaging and EE were measured in ten normal weight and ten age- and sex- matched obese participants during warm (~23 to 24 °C) and cold (~16 °C) exposure (Extended Data Fig. 3b and Table 2). Cold exposure increased EE only in the normal weight group, whereas supraclavicular skin temperature was lower in obese participants during both warm and cold conditions (Table 2).

**Table 2:** Participant data from study in normal weight and obese healthy volunteers

	Normal weight (*n* = 10)	Obese (*n* = 10)	*P* value
Age (years)	28.3 ± 2.1	28.2 ± 2.1	0.982
Sex	5 males, 5 females	4 males, 6 females	
BMI (kg m^−^^2^)	21.5 ± 0.4	36.2 ± 1.3	**4.20** **×** **10**^**−9**^
Fat mass (kg)	10.9 ± 1.1	38.1 ± 2.3	**2.45** **×** **10**^**−9**^
Fat percentage (%)	17.3 ± 2.0	35.8 ± 2.0	**3.99** **×** **10**^**−6**^
Systolic blood pressure (mmHg)	121 ± 3	129 ± 4	0.143
Diastolic blood pressure (mmHg)	72 ± 3	80 ± 3	0.069
Outdoor temperature (°C)	8.4 ± 1.4	7.3 ± 1.5	0.591
Warm room temperature (°C)	23.7 ± 0.3	23.5 ± 0.1	0.496
Cold room temperature (°C)	16.0 ± 0.2	16.1 ± 0.1	0.690
Indirect calorimetry data			
EE in warm (kcal d^−1^)	1,477 ± 81	1,897 ± 101	**0.004**
EE in cold (kcal d^−1^)	1,635 ± 85*	1,984 ± 84	**0.009** *0.008
CIT (kcal d^−1^)	158 ± 47	86 ± 52	0.322
Thermal imaging data			
Supraclavicular skin temperature in warm (°C)	35.4 ± 0.1	34.7 ± 0.1	**6.18** **×** **10**^**−4**^
Supraclavicular skin temperature in cold (°C)	34.4 ± 0.2	33.4 ± 0.2	**8.40** **×** **10**^**−4**^
Sternal temperature in warm (°C)	34.3 ± 0.2	33.6 ± 0.2	**0.028**
Sternal temperature in cold (°C)	32.7 ± 0.3	31.0 ± 0.3	**0.002**
Cold-induced change in supraclavicular temperature (°C)	-1.0 ± 0.1	-1.4 ± 0.2	0.061
Cold-induced change in sternal temperature (°C)	-1.7 ± 0.2	-2.6 ± 0.2	**0.002**
Temperature differential between supraclavicular and sternum in warm (°C)	1.1 ± 0.2	1.1 ± 0.2	0.788
Temperature differential between supraclavicular and sternum in cold (°C)	1.7 ± 0.3	2.4 ± 0.3	0.157
Cold-induced change in temperature differential (°C)	0.7 ± 0.1	1.2 ± 0.2	**0.018**

Data are mean ± s.e.m. for all 20 participants. The obese group had lower supraclavicular and sternal temperatures during both warm and cold conditions. Outdoor temperature was measured at 10 a.m. on the morning of each study visit. Comparisons between groups were performed using the unpaired *t*-test and between warm and cold exposure using the paired *t*-test. Significant *P* values are in bold. Cold exposure increased EE in the normal weight group (*P = 0.008) but not the obese group (*P* = 0.131).

Source data

During warm conditions, circulating platelet-poor serotonin concentrations were similar between normal weight and obese groups (Fig. 5b), but platelet serotonin levels were increased in obese participants (Fig. 5c). In addition, WAT serotonin levels were lower in obese participants during warm conditions (Fig. 5d). These data suggest that obesity does not increase free serotonin levels or action during warm conditions. However, cold exposure increased platelet serotonin concentrations in normal weight participants, but reduced serotonin levels in the obese group, with a concomitant increase in their WAT serotonin levels (Fig. 5c,d). Furthermore, cold exposure did not decrease platelet-poor serotonin concentrations in obese participants (Fig. 5b, interaction between room temperature and obesity *P* < 0.05). These data suggest that increased platelet serotonin uptake probably accounts for the reduction in circulating serotonin during acute mild cold exposure in normal weight individuals; however, this mechanism is dysregulated in obesity with serotonin release from platelets increasing adipose serotonin concentrations. The cold-induced rise in circulating noradrenaline concentrations was reduced in obese participants (Fig. 5e), although NEFA concentrations were similar (Fig. 5f). Insulin concentrations were substantially higher in the obese group (Fig. 5g), although glucose levels were unchanged (Fig. 5h). These data reveal dysregulation of circulating and tissue serotonin levels in obesity in response to cold.

## Discussion

**Fig. 6:**
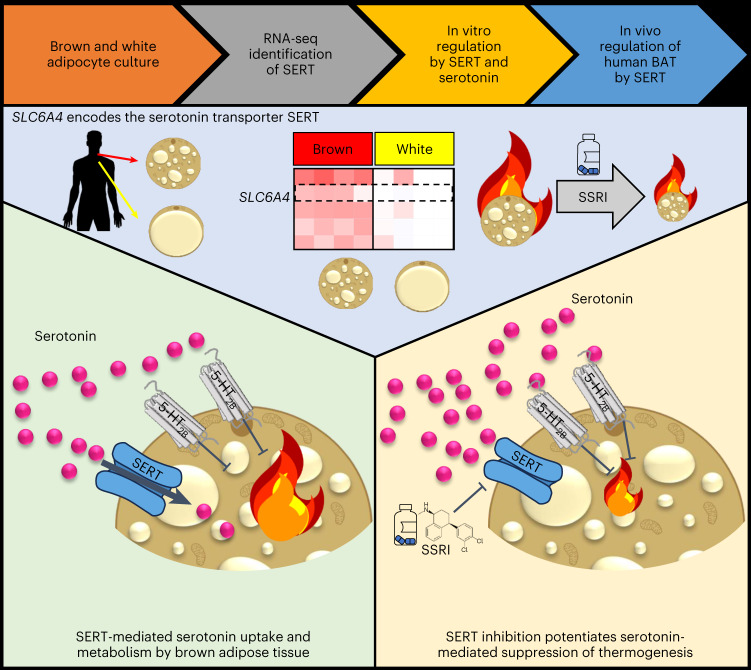
Graphical abstract depicting the role of SERT in human BAT. RNA-seq of human primary brown and white adipocytes identified the SERT as a top DEG in brown adipocytes. We have shown that serotonin suppresses brown adipocyte thermogenesis and decreases UCP1 expression through the 5-HT_2B_ receptor. We have also determined that SERT functions to scavenge extracellular serotonin in human brown adipocytes, and that inhibition of SERT by SSRIs augments the suppressive effect of serotonin on BAT thermogenesis.

We have identified the serotonergic pathway as an important regulator of human BAT thermogenesis, revealing that serotonin reduces UCP1 expression through the 5-HT_2B_ receptor to inhibit uncoupling of mitochondrial respiration (Fig. 6). We also demonstrate scavenging of extracellular serotonin by its transporter SERT in human brown adipocytes and that inhibition of SERT in vitro potentiates serotonin’s suppressive effect on brown adipocyte thermogenesis and, in vivo, reduces BAT glucose uptake and supraclavicular skin temperature during mild cold exposure. In this respect, humans differ sharply from mice, because *Slc6a4* expression was undetectable in murine brown and beige adipocytes and reduced in BAT and beige adipose tissue compared with WAT. However, serotonin also suppresses BAT thermogenesis in mice so this mechanism is preserved between species^28–30^. These data also highlight the value of using human primary brown adipocytes for translational studies of human BAT, because SERT was not enriched in previous studies using immortalized human brown adipocytes^21,22^. These findings suggest that humans may have more protection against the deleterious effects of serotonin on BAT function than mice, but also that SERT inhibition may have a greater negative impact on BAT thermogenesis. However, mice with global deletion of *Slc6a4* have greater adiposity and insulin resistance than wild-type littermates, in addition to reduced food intake and locomotor activity, probably owing to substantial central effects of SERT deletion^40–42^. *Slc6a4*^−/−^ mice also have reduced BAT mass and UCP1 levels, potentially indirectly through reduced oestradiol action^43^. *Slc6a4*^*−/−*^ mice, however, have almost no circulating serotonin because SERT is essential to transport gut-derived serotonin to the circulation^36^. Because reduced peripheral serotonin enhances BAT thermogenesis in mice^29^, this may suggest that the adverse central effects of SERT deletion on metabolic health have a greater effect than any beneficial reduction in peripheral serotonin in that model, or that complete absence of peripheral serotonin is metabolically disadvantageous. In our human study, partial SERT inhibition using sertraline decreased platelet serotonin but increased platelet-poor serotonin levels, as observed with other SSRIs^44,45^, in keeping with increased free serotonin levels and action. Although sertraline reduces SERT function in the gut and lowers total body peripheral serotonin, the reduced serotonin uptake by platelets will lead to a greater proportion of free to stored serotonin in the blood (Fig. 4e,f) and presumably increased delivery to tissues. In addition, SERT inhibition in the brown adipocytes themselves will further increase extracellular serotonin action on BAT. Therefore, the in vivo data are consistent with the hypothesis that SSRIs potentiate serotonin-mediated suppression of BAT thermogenesis as demonstrated in vitro (Fig. 2). However, more chronic sertraline use may reduce free serotonin levels^46^, although our clinical PET–CT data demonstrate that chronic SSRI treatment maintains suppression of ^18^F-FDG uptake by BAT.

SSRIs are the most commonly prescribed class of antidepressants. Unlike serotonin, they cross the blood–brain barrier to exert their therapeutic action and so inhibit SERT centrally and peripherally. SSRIs induce short-term mild weight loss, as seen in this study^34^. This effect is mediated centrally through reduced appetite and food intake via increased activation of serotonin receptors such as 5-HT_1B_ and 5-HT_2C_ (refs. ^47,48^). However, these effects on appetite are not maintained and chronic SSRI use is associated with obesity, dyslipidaemia and increased risk of diabetes^49,50^. In addition to the negative effect on BAT activity that we describe, peripheral serotonin increases hepatic gluconeogenesis and WAT lipolysis through the 5-HT_2B_ receptor in mice, whereas adipose-specific deletion of *Htr2b* protects against insulin resistance and hepatic steatosis^51,52^. Sertraline increased circulating NEFAs at baseline in our study, potentially consistent with increased serotonin-induced activation of lipolytic 5-HT_2B_ receptors in WAT, particularly as sertraline did not alter other markers of insulin resistance such as insulin or glucose concentrations. Other classes of antidepressants are also associated with obesity and metabolic disease^53^; however, most have some inhibitory action on SERT including tricyclic antidepressants, serotonin and norepinephrine reuptake inhibitors, and trazodone^54^. Interestingly, in our retrospective analysis of ^18^F-FDG-PET–CT scans, mirtazapine-treated patients accounted for 75% of the ‘BAT-positive’ participants prescribed any antidepressant medication. Mirtazapine does not inhibit SERT, which is the most likely cause of this observation; however, mirtazapine is an inhibitor of the 5-HT_2A_ receptor^55^, which could suggest a role for this receptor regulating BAT activation in vivo. Mirtazapine is also an inhibitor of the 5-HT_2c_ receptor, which may account for the weight gain observed with this medication^53^. Our results support the notion that SERT inhibition-induced suppression of BAT activity is a new mechanism through which several classes of antidepressants exert adverse metabolic effects. Although any deleterious metabolic effects of these medications are most probably outweighed by their positive effects on mental health, these data may help guide development of newer agents that retain the beneficial central effects while having a more benign metabolic risk profile.

We demonstrate that serotonin directly inhibits uncoupling of human brown adipocyte respiration and reduces UCP1 via the 5-HT_2B_ receptor. *HTR2B* is a G_q_-coupled receptor that increases intracellular calcium following activation so might be expected to increase rather than decrease BAT thermogenesis^56^. The specific mechanism through which 5-HT_2B_ mediates its inhibitory effect on human UCP1 is unclear and requires further research, but this receptor can recruit alternative signalling through β-arrestin that could suppress β-adrenergic activation, for example^56,57^. In addition, the 5-HT_2C_ receptor can also inhibit cyclic AMP production through G_i_-coupling in addition to acting through its classic G_q_ pathway^58^, but it is unclear whether the 5-HT_2B_ receptor could act through a similar mechanism in brown adipocytes. In vitro, 100 nM, but not 10 nM, serotonin supressed brown adipocyte UCP1 and cellular respiration if serotonin uptake was inhibited. The serotonin concentration noted in platelet-poor plasma in humans (~20 nM) would be sufficient to activate the 5-HT_2B_ receptor in vivo^59^, although we were unable to measure interstitial serotonin concentrations in BAT in our volunteers. Although serotonin exerts its biological effects primarily extracellularly via activation of its receptors, following uptake serotonin’s metabolites can also mediate effects intracellularly^60^. However, our data suggest this is mediated through an extracellular mechanism, because preventing serotonin uptake through sertraline augmented serotonin’s suppressive effect. SSRIs may exert some of their in vivo effects on BAT indirectly, either centrally or through other peripheral tissues. For example, acute SSRI administration decreases basal noradrenaline appearance rates, a measure of sympathetic tone^61^, so could reduce cold-induced sympathetic activation. In our study, sertraline reduced circulating noradrenaline concentrations during warm exposure, but did not suppress the cold-induced rise in noradrenaline and NEFAs. Furthermore, sertraline did not alter skeletal muscle ^18^F-FDG uptake or substantially alter the increase in EE (~35 kcal per 24 h lower on sertraline phase) during cold exposure, in keeping with a selective effect on BAT^62^. Sertraline also reduced ^18^F-FDG uptake by BAT similarly in male and female participants which does not support any indirect oestrogen-mediated effect in this group^43^.

The substantial reduction (~40%) in circulating serotonin concentrations during acute mild cold exposure in ‘BAT-positive’ healthy volunteers was a surprising finding. This will probably reduce serotonin delivery to tissues such as BAT and, because serotonin suppresses brown adipocyte thermogenesis, could represent a new physiological cold-induced mechanism that enhances BAT thermogenesis. We demonstrate that this reduction in circulating serotonin is paralleled by increased serotonin concentrations in platelets; this is most probably due to enhanced removal of circulating serotonin by platelets that highly express SERT^63^. A potential mediator of this effect is noradrenaline, which increases serotonin uptake by platelets^64^. Human obesity has previously been associated with increased circulating serotonin concentrations^39^ in addition to polymorphisms in *TPH1*, the gene that encodes the rate-limiting enzyme in peripheral serotonin synthesis^65^. However, it was the platelet rather than platelet-poor serotonin concentrations that were increased in our obese volunteers, suggesting that free serotonin levels are not increased in obesity. Cold exposure reduced platelet serotonin in obese participants, in parallel with an increase in adipose serotonin, highlighting dysregulation of this response. Although room temperatures were similar between groups, the cold exposure may have been insufficient to elicit a similar response in the obese compared with the normal weight participants, most probably owing to their greater basal EE (Table 2). Therefore, more severe cold exposure which increased EE to a similar extent in obese and normal weight groups may have changed the response of circulating and tissue serotonin levels particularly in the obese group. However, this cold temperature was sufficient to increase circulating noradrenaline and NEFA levels in obese study participants and crucially platelet/adipose serotonin levels decreased/increased respectively during cold rather than remaining unaltered. Our data demonstrate that the peripheral serotonergic system is an important regulator of human BAT. Modulation of this pathway has been previously proposed as a new treatment for obesity and metabolic disease. Possible therapeutic options include inhibition of peripheral serotonin synthesis using TPH1 inhibitors^28,29^ and peripheral 5-HT_2B_ receptor antagonists^51,52^, targets recently identified in mice. Although SERT expression may provide some protection for human BAT against peripheral serotonin, this would not protect other important metabolic organs such as muscle and WAT.

Our study has several limitations in addition to those already discussed. Although we showed that serotonin and SSRIs reduce BAT activation in vitro, we did not directly measure BAT thermogenesis in vivo because we used ^18^F-FDG as a marker of BAT activation. Glucose uptake by BAT increases during cold exposure and adrenergic stimulation^18^, correlates with BAT temperature as measured by proton magnetic resonance spectroscopy^66^ and with CIT during similar mild cold exposure^35^, but dietary interventions^67^ and diabetes^68^ can reduce glucose uptake by BAT without impacting oxidative metabolism. Although we did not alter the diet of these study participants and sertraline did not alter insulin and glucose levels, changes in glucose uptake may not always reflect thermogenesis. The change in ^18^F-FDG uptake by BAT correlated with the change in EE between the sertraline and placebo phases, and sertraline induced an appropriate reduction in supraclavicular skin temperature. Although these data could be interpreted as measurements consistent with a reduction in BAT thermogenesis, thermal imaging measures skin temperature so is not a specific measurement of BAT thermogenesis and can be confounded by changes in local blood flow, skin thickness and heat production by other tissues such as muscle. Although CIT was not different between SSRI and placebo phases, it is also possible that sertraline alters the body’s perception of cold stress (altering the set point of BAT activation) rather than specifically inhibiting BAT thermogenesis. Therefore, it is possible that more severe cold exposure may have increased ^18^F-FDG uptake by BAT further on the SSRI-treated phase. In addition, we only measured the BAT fat fraction following mild cold exposure so could not determine whether sertraline increased the fat fraction in these study participants before or during BAT activation. Therefore, it is possible that sertraline increases BAT triglyceride content during warm exposure rather than inhibiting BAT lipolysis during cooling. We also did not undertake PET scanning in the case-control study so cannot determine the prevalence of BAT in those individuals. Furthermore, we were unable to quantify interstitial serotonin concentrations in BAT in these study participants, which would have necessitated a technique such as microdialysis to determine whether these levels are dysregulated in obesity^35^, because adipose tissue biopsies may not reflect extracellular concentrations.

To conclude, we have identified the serotonin transporter as an important regulator of human BAT function, taking this candidate from ‘bench to bedside’. These data reveal new species-specific differences in the regulation of BAT function and a new mechanism protecting human BAT from serotonin-mediated suppression of BAT activation. Finally, these data identify a candidate mechanism through which chronic administration of SSRIs and other antidepressants cause weight gain and metabolic dysfunction. Further research is required to assess the therapeutic benefit of reducing peripheral serotonin action as a new strategy to treat metabolic disease.

## Methods

### In vivo crossover study in healthy volunteers

Fifteen healthy volunteers aged 18–35 years (Table 1) were recruited to a double-blind placebo-controlled randomised crossover study. Inclusion criteria were as follows: BMI 18.5–25 kg m^−^^2^; weight change of <5% in preceding 6 months; no acute or chronic medical conditions; on no regular medications; alcohol intake ≤14 units per week; no claustrophobia or contraindication to MRI scanning; no pregnancy or breastfeeding in female participants; normal screening blood tests (full blood count, glucose, kidney, liver and thyroid function). Approval was obtained from the South East Scotland Research Ethics Committee (ethics number 18/SS/0104) and informed consent was obtained from each study participant. Volunteers received the SSRI sertraline at a dose of 50 mg daily for 7 days or placebo in randomised order (Extended Data Fig. 3a). Sertraline was chosen as the SSRI for this study because it demonstrates minimal inhibition of the noradrenaline transporter^69^ and achieves high concentrations in adipose tissue^70^. On the seventh day of tablet administration, study participants attended the Edinburgh Clinical Research Facility (ECRF) in the Royal Infirmary of Edinburgh at 08:00 hours following an overnight fast. Volunteers were instructed to avoid alcohol for 7 days and exercise for 2 days before each visit and to wear identical standard light identical clothing at each visit. At each study visit, measurements of height, weight, body fat mass by bioimpedance (using an Omron BF302 monitor) and blood pressure were performed. Fasting blood samples were obtained for measurement of glucose, insulin, NEFAs and noradrenaline. At time *T* = 0 min, participants were placed in a room at 23–24 °C (warm room) for 1 h (Extended Data Fig. 3a). At *T* + 60 min, volunteers were moved to a room cooled to 16–17 °C (cold room) for 3 h (up to *T* + 240 min) to activate their BAT. Participants were checked every 15 min for signs or symptoms of shivering. Blood samples were obtained every 30 min from *T* = 0 to *T* + 120 min, EE was measured twice each hour over the same period. Thermal imaging was performed of the upper body region intermittently from *T* = 0 to *T* + 180 min to measure supraclavicular and lower sternal skin temperatures. At *T* + 180 min, participants received an intravenous injection of 75 MBq of ^18^F-FDG. At *T* + 240 min, participants underwent a PET–MRI scan to quantify BAT mass and activity. Following the scan, volunteers returned home. Female participants attended for their second visit 4 or 8 weeks after their initial visit to ensure they were in identical phases of their menstrual cycle. Male participants attended their second visit at least 3 weeks after the first.

### In vivo study in normal weight and obese healthy volunteers

Twenty healthy volunteers aged 18–40 years (Table 2) were recruited to a case-control study. Inclusion criteria were as follows: BMI 18.5–25 kg m^−^^2^ (normal weight group) or 30–55 kg m^−^^2^ (obese group); weight change of <5% in preceding 6 months; no acute or chronic medical conditions known to alter BAT activity or preclude an abdominal adipose tissue biopsy; on no regular medications known to alter serotonin concentrations or BAT activity; alcohol intake ≤14 units per week; no pregnancy or breastfeeding in female participants; no allergy to local anaesthetic; normal screening blood tests (full blood count, glucose, kidney, liver and thyroid function). Approval was obtained from the Edinburgh Medical School Research Ethics Committee (ethics number 18-HV-049) and informed consent was obtained from each participant. Subjects attended the ECRF at 0800 hours following an overnight fast. Volunteers were instructed to avoid alcohol and exercise for 2 days before the study visit. Measurements of height, weight, body fat mass by bioimpedance and blood pressure were performed. Blood samples were obtained for measurement of glucose, insulin, NEFAs, serotonin and noradrenaline. At *T* = 0 min participants were placed in a room at 23–24 °C (warm room) for 2 h (Extended Data Fig. 3b). At *T* + 120 min, volunteers were moved to a room cooled to 16–17 °C (cold room) for 2 h (up to *T* + 240 min). Participants were checked every 15 min for signs or symptoms of shivering. Blood samples were obtained every 30 min from *T* = 0 to *T* + 240 min, EE was measured once each hour over the same period. Thermal imaging was performed of the upper body region intermittently from *T* = 0 to *T* + 240 min to measure supraclavicular and lower sternal skin temperatures. At *T* + 105 and *T* + 225 min (following 105 min of warm and cold exposure), an abdominal subcutaneous adipose tissue biopsy was performed. Following the final blood sample, volunteers were given lunch and allowed home.

### PET–MR scanning protocol and analysis

All participants received 75 MBq of ^18^F-FDG 1 h before being placed supine on a Siemens mMR scanner (Siemens Healthineers). Following initial localization, a standard MRAC_GRAPPA scan was acquired for each bed position, used to calculate a standard umap for attenuation correction. A three-dimensional T1-weighted Dixon VIBE acquisition was used to generate images at 1.34 and 2.56 ms (repetition time (TR) = 4.02) to calculate a fat fraction map (FFM) as outlined below. ^18^F-FDG uptake by BAT was quantified using Analyze v.12.0 (AnalyzeDirect). We developed a semi-automated process to standardize the PET/MRI analyses. Following registration, the MRI fat and water images were used to generate a FFM of each participant using the following equation:

$${\mathrm{F{at}}}\,{{\mathrm{fraction}}}\,( \% )\,=\frac{\mathrm{Signal}\,\mathrm{intensities}\,\left(\mathrm{SI}\right)\,\mathrm{fat}}{\,\mathrm{SI}\,\mathrm{fat}+\mathrm{SI}\,\mathrm{water}}\,\times \,100$$

A median spatial filter was applied to the FFM, and any voxels below 50% fat fraction (that is not adipose tissue) were removed to generate a fat fraction region of interest (ROI). The registered PET images were loaded and thresholded using the BARCIST criteria (a standard uptake value (SUV)_lean_ of ≥1.2 g ml^−1^ for each participant^9^). Any remaining ROIs that encompassed the brain were manually removed. Voxels meeting both the FFM and PET thresholds were analysed. Cervical, supraclavicular and axillary depots were tagged together (classified as supraclavicular or SCV for simplicity), whereas paraspinal BAT was tagged separately. Finally, image erosion with a (3 × 3 × 1) structuring element was applied to the BAT ROIs to remove PET blooming and boundary artefacts. The effect of sertraline on ^18^F-FDG uptake by BAT was similar irrespective of whether image erosion was performed (see Source data for Fig. 3 and Extended Data Fig. 4). Data are presented for the BAT SUV_mean_ and BAT volume, in addition to total ^18^F-FDG uptake by BAT (SUV_mean_ multiplied by BAT volume) as previously described^20^.

For skeletal muscle analysis, ROIs from the pectoralis major, psoas major, sternocleidomastoid, longus colli and trapezius muscles, and in abdominal subcutaneous adipose tissue, were drawn manually using the MR water image, ensuring ROIs were identically sized and positioned at both study visits. These ROIs were applied to the registered and filtered PET images as described above for analysis.

### Thermal imaging

Thermal imaging was performed as described previously^20^. In brief, thermal images of the volunteers’ upper body were obtained using a FLIR T650sc camera at defined intervals (generally 10–15 min apart during specific procedures such as adipose tissue biopsies). The camera was positioned 1 m from the participant and the emissivity set to 0.98 for human skin. Identically sized regions of interest were drawn around the left and right supraclavicular regions and the lower sternal region (as demonstrated in Fig. 3c) using FLIR Tools (FLIR). The mean and maximum supraclavicular (left and right) and lower sternal temperatures were recorded from each image. Data are presented as the mean of the mean supraclavicular and sternal temperatures recorded during warm and cold conditions.

### Indirect calorimetry

EE was measured for 15 min on two occasions during both warm and mild cold exposure using a ventilated-hood indirect calorimeter (GEM Nutrition). The first 5 min of data were discarded and the mean value over the final 10 min was recorded. EE is presented as the mean of the two values obtained during warm and cold exposure. CIT was calculated by subtracting the mean EE obtained in the warm room from the EE measured in the cold room as previously described^20,35^.

### Abdominal adipose tissue biopsy

Anterior subcutaneous adipose tissue biopsies were performed by needle aspiration as described previously^71^. Following sterilization of the area and injection of 5 ml 2% lidocaine, a 14 G needle and 50 ml syringe was inserted ~5 cm lateral to the umbilicus. Following aspiration of ~200 mg adipose tissue, samples were washed with 0.1% diethylpyrocarbonate-treated water then immediately frozen on dry ice before storing at −80 °C. The biopsy obtained during cold exposure was aspirated from the contralateral abdominal depot to the one taken while in the warm room.

### Biochemical assays

Plasma serotonin and noradrenaline (both LDN) and serum insulin (Mercodia) were measured by enzyme-linked immunosorbent assay (ELISA). Colorimetric assays were used to measure plasma glucose (Sigma-Aldrich) and NEFAs (Wako Diagnostics). For measurement of serotonin concentrations in platelet-poor and platelet fractions, EDTA plasma samples were subjected to centrifugation at either 2,000*g* (platelet-poor plasma) or 200*g* (platelet fraction) for 10 min at room temperature. The platelet fraction was further prepared by adding five volumes of saline to the supernatant which was then spun at 4,500*g* for 10 min at 4 °C; finally the pellet was resuspended in water. Difficulties in blood sampling in two participants on one phase of the SSRI study and during cold exposure in three participants in the case-control study led to platelet rupture meaning we were unable to measure platelet-poor or platelet serotonin levels in these volunteers, hence data are reported for the remaining 13 and 17 participants respectively. Screening blood tests were analysed by the Royal Infirmary of Edinburgh biochemical laboratory for serum kidney, liver and thyroid function and plasma glucose using an Abbott ARCHITECT c16000 analyser and for full blood count using a Sysmex XE-5000 analyser.

### Analysis of circulating sertraline concentrations by liquid chromatography tandem mass spectrometry

Lithium heparin plasma samples were obtained after overnight fast during both phases following 7 days of sertraline and placebo tablets. Targeted analysis of sertraline and its major metabolite norsertraline was carried out by automated supported liquid extraction followed by liquid chromatography tandem mass spectrometry in multiple reaction mode. Calibration standards were prepared ahead of time by enriching blank human plasma with sertraline and norsertraline (N-049 and S-021 respectively; Cerilliant) across expected ranges (0.25–50 and 2.5–100 ng ml^−1^, respectively). On the day of extraction, 200 μl of standard and samples were aliquoted into 96-well deep-well plates, enriched with 1 ng of d3-sertraline (S-026; Cerilliant) as an internal standard. These were diluted on an Extrahera liquid extraction robot (Biotage) with 0.5 M ammonium hydroxide (200 μl), on an SLE400 plate, followed by elution with ethyl acetate (1,800 μl), reduction to dryness, resuspension in water/methanol (100 μl; 90:10 v/v water/acetonitrile), and the plate was sealed with zone-free 96-well plate sealing film (Sigma-Aldrich) before liquid chromatography tandem mass spectrometry analysis.

Liquid chromatographic separation was achieved by injection (10 μl) on to an Acquity I-Class UPLC (Waters) using a Kinetex C18 column (150 × 2.1 mm; 2.6 μm; Phenomenex), protected by a Kinetex KrudKatcher (Phenomenex) at 40 °C. The mobile phase consisted of 0.1% formic acid in water (A) and 0.1% formic acid in acetonitrile (B) at a flow rate of 0.4 ml min^−1^. Gradient elution was achieved with a total run time of 12 min from 20% to 90% B. The analytes were detected on a QTrap 6500+ mass spectrometer (AB Sciex) operated in positive electrospray mode (500 °C, 4.5 kV). The analytes and internal standard sertraline, d3-sertraline and norsertraline eluted from the column at 4.90, 4.89 and 4.75 min respectively. Multiple reaction mode transitions monitored for were sertraline *m/z* 306.1 → 159.0, 275.0 at 31 and 15 V, norsertraline *m/z* 275.0 → 159.0, 129.0 at 21 and 19 V and d3-sertraline *m/z* 309.1 → 159.0 at 16 V.

Linear regression analysis was applied to the ratio of the peak area of sertraline (1/*x*^2^) and norsertraline (1/*x*) to the internal standard d3-sertraline using Analyst software, and the amount of sertraline and norsertraline in the samples was calculated from the peak area ratio using the linear regression analysis equation.

### Analysis of adipose tissue serotonin concentrations

Adipose tissue was homogenized in 0.2 N perchloric acid and subject to centrifugation at 10,000*g* for 5 min at 4 °C. The collected supernatant was mixed 1:1 in 1 M borate buffer (1 M boric acid, pH 9.25) and spun at 10,000*g* for 1 min. The supernatant was collected and stored at −80 °C until analysis. Serotonin concentrations were determined by ELISA (Beckman Coulter).

### Analysis of retrospective ^18^F-FDG-PET–CT patient scans

Local Caldicott guardian approval was obtained. Patients were included who received scans between two separate periods, from December 2014 to January 2016 and from November 2017 to June 2019. Participants were assigned into three groups: (1) those currently prescribed an SSRI, (2) those currently prescribed any other class of antidepressant, and (3) those not currently prescribed antidepressants (Extended Data Table 2). Groups were matched for sex, age, body weight, fasting glucose, presence of diabetes, underlying cancer diagnosis and outdoor temperature in the month of scan. Patients received either a fixed a dose of 370 MBq or 4 MBq kg^−1^ of ^18^F-FDG 1 h before being placed supine in a hybrid PET–CT scanner. Two models of hybrid PET–CT scanners were used, a Biograph mCT (Siemens Medical Systems) or a Discovery 710 (GE Healthcare). All scans were performed at room temperature and participants underwent a low-dose CT for attenuation correction (non-enhanced, 120 kV) with tube current modulation applied (50 mAs quality reference) followed by static PET imaging of the upper body using 3-min beds. On the Biograph, the data were reconstructed using Siemens UltraHD reconstruction (2 iterations and 21 subsets), on the Discovery 710 the QClear reconstruction was used with a beta value of 400. Images were analysed using PMOD v.3.7 (PMOD Technologies). ^18^F-FDG uptake by BAT was quantified by measuring the mean SUV from all pixels with an SUV of >1.5 g ml^−1^ normalized to body mass, which corresponded to tissues with a radio density on the CT scan with Hounsfield units between −190 and −10^9^. Participants were classified into ‘BAT-positive’ or ‘BAT-negative’ based on the above analysis. The mean SUV and the volume of BAT with detectable ^18^F-FDG uptake were calculated for the BAT-positive patients.

### Effect of cold exposure on circulating serotonin levels in BAT-positive participants

Stored blood samples obtained from 11 male study participants with detectable ^18^F-FDG uptake by BAT during cold exposure^20,35^ were analysed to determine whether acute cold exposure altered circulating serotonin concentrations. Plasma samples were analysed from these participants following <2 h of warm exposure at ~24 °C and following approximately 60 min of exposure to mild cold at ~17 °C. Serotonin concentrations were measured by ELISA analysis (LDN) as described above.

### In vitro studies on primary adipocytes

#### Tissue collections

Male and female euthyroid participants were recruited (all participant data is given in Supplementary Table 1) who were due to undergo elective thyroid or parathyroid surgery in the Royal Infirmary of Edinburgh. Local ethical approval was obtained (ethics numbers 15/ES/0094 and 20/ES/0061) as was consent from each participant. Adipose tissue was obtained intra-operatively from the central compartment of the neck, superior to the clavicle and deep to the lateral thyroid lobe either adjacent to the longus colli muscle or to the oesophagus (BAT), and more superficially from the subcutaneous neck tissue (WAT). Tissue collections were all performed by the same surgeon. Tissue samples were either immediately frozen at −80 °C for qPCR, fixed for immunohistochemistry, or the stromal vascular fraction was isolated and cultured for the respective experiments detailed below.

For murine tissue collections, male 129/Ola mice were housed at 20–21 °C and a humidity of 50–55%, using a 12 h/12 h light/dark cycle and with food and water available ad libitum. Animals were killed at 8–9 weeks of age and their interscapular BAT, inguinal and epididymal WAT were immediately collected and either frozen at −80 °C for qPCR or their stromal vascular fraction was isolated and cultured.

#### Cell culture

Cells were cultured as described previously^20^. Following collection, adipose tissue samples were incubated in Krebs–Henseleit buffer containing 0.2% collagenase type 1 at 37 °C for 45 min. Samples were subject to centrifugation at 800*g* for 10 min, the pellet resuspended and passed through a 100-μm filter, subject to centrifugation at 200*g* for 5 min and the pellet resuspended in DMEM containing 10% FBS and cultured in six-well plates. Cells were passaged when 80% confluent and differentiated in DMEM containing 10% FBS with the addition of 1 nM tri-iodothyronine, 20 nM insulin, 500 μM IBMX, 500 nM dexamethasone and 125 μM indomethacin for 7 days for human cells and 5 days for murine cells. Cells were then cultured for a further 7 days in DMEM medium containing 10% FBS, 1 nM tri-iodothyronine and 20 nM insulin before experiments.

#### Quantification of serotonin uptake by primary human adipocytes

For all the experiments involving addition of serotonin to medium, medium and samples were protected from light to prevent rapid degradation of serotonin. Cellular serotonin uptake was measured in duplicate by adapting a previously published protocol^72^. Paired white and brown adipocytes were pre-incubated for 1 h in DMEM medium containing either vehicle or ascending doses of sertraline (1 nM to 10 µM). Wells were then washed twice with uptake buffer (120 mM NaCl, 5 mM KCl, 1.2 mM CaCl_2_, 1.2 mM MgSO_4_, 1 mM ascorbic acid, 25 mM HEPES, 5 mM glucose; pH 7.4) before incubation with 20 nM [^3^H]-5-hydroxytryptamine (Perkin Elmer) and either vehicle or sertraline (1 nM to 10 µM) for 1 h. Serotonin uptake was terminated by aspiration of medium followed by three washes with uptake buffer. Cells were lysed by the addition of 1% SDS followed by a 30-min incubation at 37 °C. Wells were scraped and lysates were added to the scintillation vials containing Opti-Fluor (Perkin Elmer) for quantification by liquid scintillation counting.

#### Serotonin regulation of *UCP1* mRNA levels in human adipocytes

The serotonin concentration in DMEM medium containing 10% FBS was quantified by ELISA and measured ~410 nM. Therefore, for experiments where serotonin was added to culture medium, serotonin was stripped by incubating FBS with dextran-coated charcoal^73^, which successfully reduced the serotonin concentration in culture medium to ~3 nM. Paired white and brown adipocytes were incubated in DMEM medium containing stripped serum and insulin/tri-iodothyronine (T3) for 24 h. Thereafter, wells were incubated with medium containing either vehicle or 10 nM to 100 µM serotonin for 24 h before cell lysis for qPCR. In a separate experiment, following incubation with stripped serum medium for 24 h, white and brown adipocytes were incubated with medium containing vehicle, sertraline (1 μM), or sertraline in the presence of serotonin (10 nM to 10 μM) for 24 h.

#### Inhibition of 5-HT_2A/B_ receptor on *UCP1* mRNA levels in human brown adipocytes

Brown adipocytes were incubated in DMEM medium containing stripped serum and insulin/T3 for 24 h. Thereafter, wells were incubated with the above medium with the addition of either vehicle or 100 µM serotonin for 24 h; wells receiving serotonin were further treated with either vehicle, the 5-HT_2A_ receptor inverse agonist pimavanserin (10 µM)^74^, the 5-HT_2B_ receptor antagonist SB-204741 (10 µM)^75^ or both compounds. Thereafter, cells were lysed for qPCR analysis.

#### Knockdown of *HTR2A* and *HTR2B* in human brown adipocytes

siRNA-mediated knockdown of the 5-HT_2A/2B_ receptors was undertaken based on a previously published protocol^76^. Following differentiation, human primary brown adipocytes were transfected with 20 nM On-TARGETplus Smartpool siRNA (Dharmacon) either targeting *HTR2A* (L-005638-00-0005), *HTR2B* (L-005639-02-0005) or a non-targeting pool (D-001810-10-05), using Lipofectamine RNAimax reagent (Thermo Fisher Scientific) according to the manufacturer’s instructions. After 48 h, cells were washed with PBS buffer and replaced with medium containing stripped serum for 24 h. Thereafter, wells were incubated with this medium containing either vehicle or 100 µM serotonin for a further 24 h. Cells were then lysed in QIAzol (Qiagen) for RNA.

#### RNA extraction and quantitative real-time PCR measurements

Whole adipose tissue was homogenized in QIAzol reagent using a TissueLyser (Qiagen). mRNA was extracted from both tissue and cells using the RNeasy Lipid Kit (Qiagen) and complementary DNA was generated using the Qiagen QuantiTect reverse transcription kit. qPCR was performed in triplicate using a Roche Lightcycler 480, using gene-specific primers (Invitrogen) and fluorescent probes from the Roche Universal Probe Library, or with Taqman assays as detailed in Extended Data Table 4. Transcript levels are presented as the ratio of the abundance of the gene of interest: mean of abundance of control genes (*PPIA* and *RNA18S5* in humans, *Tbp* and *Rn18s* in mice).

#### RNA-seq of human primary adipocytes

Paired brown and white human primary adipocytes were incubated in DMEM containing 10% stripped FBS, 1 nM T3 and 20 nM insulin for 48 h. Cells were lysed and mRNA extracted as above. Before transcriptomics, *UCP1* mRNA levels were confirmed to be substantially higher in the brown adipocytes (see Source data for Fig. 1; *P* < 0.01 versus white adipocytes). RNA-seq was performed by the ECRF Genetics Core. Total-RNA quality and integrity were assessed on an Agilent Bioanalyser using the RNA 6000 Nano kit. Samples were DNase-treated using the TURBO DNA-free kit (Thermo Fisher Scientific), then quantified using the Qubit 2.0 fluorometer and the Qubit RNA BR assay, and assessed for residual DNA contamination using the Qubit dsDNA HS assay.

##### Nucleic acid library construction protocol

TURBO DNase-treated total-RNA samples were used to generate the libraries using the TruSeq Stranded Total-RNA with Ribo-Zero kit (catalogue no. RS-122-2201). Total-RNA (100 ng) was depleted of ribosomal RNA before purification, fragmentation and were primed using random hexamers. The RNA fragments were reverse transcribed using reverse transcriptase and random primers. Double-stranded cDNA was synthesized following removal of RNA templates using a replacement strand incorporating dUTP in place of dTTP. Blunt-ended cDNA was generated using AMPure XP beads (Beckman Coulter) to separate the double-stranded cDNA from the second strand reaction mix, before the addition of an ‘A’ nucleotide to 3′ ends of the blunt fragments (and a ‘T’ on the dapter 3′ end). Thereafter, multiple indexing adaptors were ligated to the double-stranded cDNA for hybridization onto a flow cell, before enrichment and amplification of adaptor-containing DNA fragments using 15 cycles of PCR to ensure the library was suitable for sequencing. Libraries were quantified using the Qubit dsDNA HS kit and by PCR using the Kapa Universal Illumina Library Quantification kit. An Agilent Bioanalyser was used for quality control.

##### Nucleic acid sequencing protocol and analysis of RNA-seq data

Sequencing was performed using the NextSeq 500/550 High-Output v2 (150 cycle) Kit on the NextSeq 550 platform. The Illumina RNA-Seq Alignment Workflow was used to generate count files (genome assembly was performed (hg19; Illumina)). Gene expression level normalization was performed by DESeq2 (v.1.26.0, Bioconductor v.3.10)^77^ which was used for downstream analysis. Pathway analysis of gene identifiers extracted from RNA-seq was performed using Ingenuity Pathway Analysis (Ingenuity Systems, www.ingenuity.com, Qiagen). Volcano plots and heat maps to visualize significantly differentially expressed transcripts (adjusted *P* value < 0.05, log_2_(fold change) > 2) were generated using Prism v.9 (GraphPad). RNA-seq data generated this study have been uploaded to ArrayExpress (E-MTAB-101123; details in Source data for Fig. 1).

#### Respirometry

Pre-adipocytes from human WAT and BAT were plated in Seahorse XF24 V7 PS cell culture plates (Agilent Technologies) and differentiated as above. White and brown adipocytes were cultured in serum-stripped medium for 24 h, and then cultured in serum-stripped medium containing either vehicle or serotonin (10 nM to 100 μM) for 24 h. In a separate experiment, following culture in serum-stripped medium for 24 h, white and brown adipocytes were cultured in serum-stripped medium containing either vehicle, sertraline (1 μM) or sertraline in the presence of serotonin (10 nM to 10 μM) for 24 h. Cells were analysed on a Seahorse XFe24 analyser as described previously^20^. Measurements were performed during basal respiration and following sequential addition of noradrenaline (2 μM), oligomycin (2.5 μM), carbonyl cyanide-*p*-trifluoromethoxyphenylhydrazone (FCCP, 2 μM) and rotenone (0.2 μM)/antimycin A (2.5 μM). The oxygen consumption rate (OCR) was calculated following subtraction of non-mitochondrial respiration. Basal respiration was calculated by taking the mean of three cycles, whereas stimulated respiration was measured using the results of the sixth cycle following the addition of noradrenaline. Uncoupled respiration was calculated by taking the mean of three cycles following addition of oligomycin, whereas maximal respiration was measured after the addition of FCCP for one cycle.

#### Immunohistochemistry

Human WAT and BAT were fixed for 24 h in 10% formalin before embedding in paraffin. Tissue sections were incubated with mouse anti-human SERT antibody (1:1,500 dilution; Millipore) or rabbit anti-human UCP1 antibody (1:8,000 dilution; Sigma-Aldrich, catalogue no. U6382) as the primary antibodies, and goat anti-mouse or goat anti-rabbit (both 1:200 dilution; Vector Laboratories) as the secondary antibodies following the avidin–biotin complex method as described previously^20^. Antigen retrieval was conducted in Tris-EDTA buffer (pH 9) for 5 min in a pressure cooker. Images were taken on a NIKON Eclipse Ci-L mounted with a NIKON DS-Fi3 camera and DS-L4 controller set.

### Statistical analyses

Data are presented as mean ± s.e.m. All analyses (except RNA-seq, detailed above) were performed either using Prism software v.9 (GraphPad) or SPSS v.25 (IBM). A *P* value <0.05 was considered statistically significant and all tests performed were two-sided. The group numbers in the figure captions indicate the number of independent biological replicates. Data were analysed for normal distribution within each experimental group. Comparisons between two related groups were examined using the paired *t*-test, whereas comparisons between two unrelated groups were performed by unpaired *t*-test. Comparisons involving three or more related groups were analysed using two-way repeated measures analysis of variance (ANOVA) with post-hoc testing. Where data was not normally distributed, for comparisons between two related groups the Wilcoxon signed-rank test was used. Associations were tested using Pearson’s correlation coefficient. The specific statistical tests used are detailed in the respective figure captions along with the number of biologically independent samples used for each comparison. All data generated or analysed during this study are included in this published article (including supplementary information, extended data figures, tables and source data).

### Reporting summary

Further information on research design is available in the Nature Portfolio Reporting Summary linked to this article.

### Supplementary information

Supplementary Information Supplementary Table 1.

Reporting Summary

### Source data

Source data for Figs. 1–5, Tables 1 and 2 and Extended Data Figs. 1–4.

## Figures and Tables

**Extended Data Table 1:**
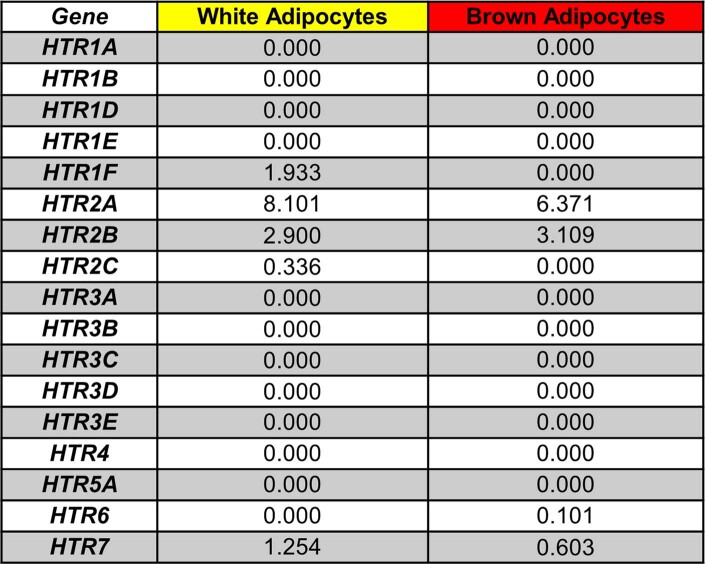
Serotonin receptor expression from RNA sequencing data

Transcript expression values of the various 5-HT receptor subtypes in paired primary human brown and white adipocytes (n = 4/ group). The 5HT_2_ receptors were the most highly expressed in both cell types.

**Extended Data Table 2:**
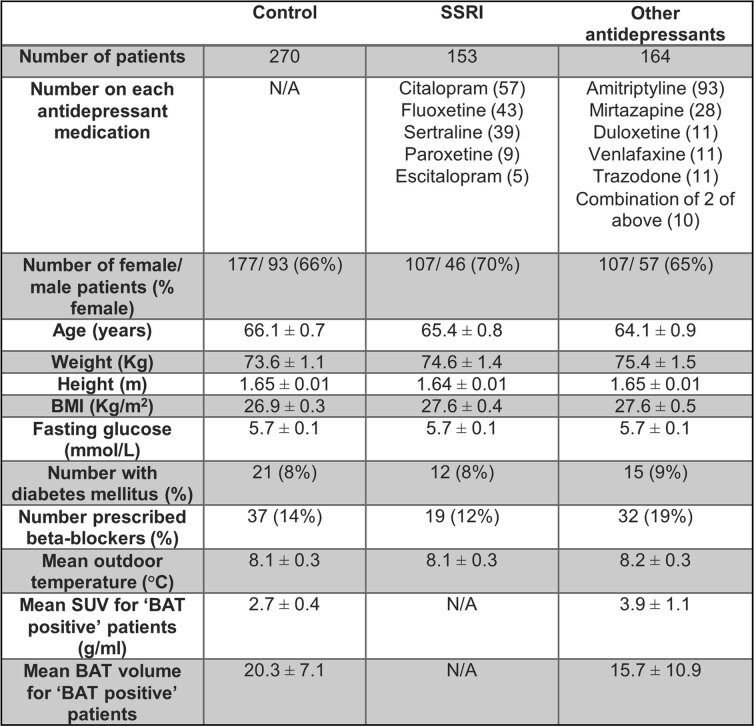
Patient characteristics from retrospective analysis of PET/CT scans

Data are mean ± SEM. Anthropometric and biochemical data from patients who attended the Royal Infirmary of Edinburgh to undergo PET/CT scanning as part of their diagnostic pathway. All patients received either 370MBq or 4MBq/Kg of ^18^F-FDG 60 minutes prior to undergoing PET/CT scanning of their body. Patients were kept in a room at 20-21˚C prior to and following the ^18^F-FDG injection until the PET/CT scan was performed. Data were analysed by one way ANOVA or by Chi-squared test, there were no statistically significant differences between groups. Historical ambient temperature data were obtained from the Met Office Leuchars station which is situated closest to Edinburgh, Scotland, United Kingdom (https://www.metoffice.gov.uk/pub/data/weather/uk/climate/stationdata/leucharsdata.txt).

**Extended Data Table 3:**
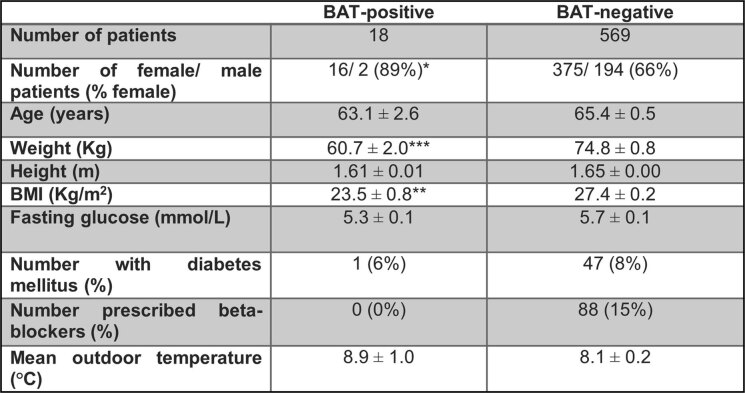
Patient characteristics with detectable ^18^F-FDG uptake by BAT

Data are mean ± SEM. Anthropometric and biochemical data from patients who attended the Royal Infirmary of Edinburgh to undergo PET/CT scanning as part of their diagnostic pathway. All patients received either 370MBq or 4MBq/Kg of ^18^F-FDG 60 minutes prior to undergoing PET/CT scanning of their body. Patients were kept in a room at 20-21˚C prior to and following the ^18^F-FDG injection until the PET/CT scan was performed. Patients were divided into those with (BAT-positive) and without (BAT-negative) detectable ^18^F-FDG uptake by BAT. Data were analysed by unpaired t test, Mann Whitney U test or by Chi-squared test. Historical ambient temperature data were obtained from the Met Office Leuchars station which is situated closest to Edinburgh, Scotland, United Kingdom (https://www.metoffice.gov.uk/pub/data/weather/uk/climate/stationdata/leucharsdata.txt).*P = 0.042, **P = 0.002, ***P < 0.001 vs BAT negative group. Beta-blocker use tended (P = 0.07) to be associated with the BAT negative group.

**Extended Data Table 4:**
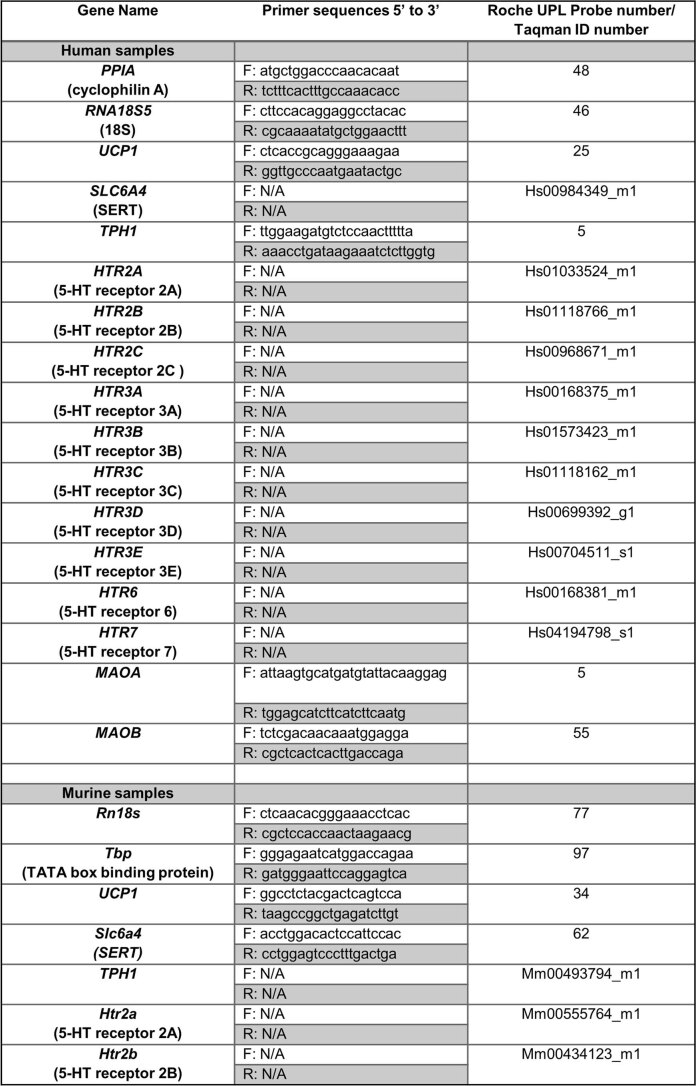
Primer sequences for qPCR and corresponding probe numbers

Assays were either performed using the following primer sequences and the relevant Roche probe library or using the following Taqman assay ID numbers.

## Data Availability

The data that support the findings of this study are available in the accompanying Source data while the transcriptomics data are available at ArrayExpress (E-MAB-101123) E-MTAB-10123 < ArrayExpress < BioStudies < EMBL-EBI.
