# Supplementary material for: The serotonin transporter sustains human brown adipose tissue thermogenesis
Source: Nat Metab. 2023 Aug 3;5(8):1319–36. doi: 10.1038/s42255-023-00839-2 (PMC10447248; doi:10.1038/s42255-023-00839-2)
Supplement: Supplementary file 1 — Supplementary Table 1. [file 42255_2023_839_MOESM1_ESM.pdf]

nature metabolism

Article https://doi.org/10.1038/s42255-023-00839-2

The serotonin transporter sustains human brown adipose tissue thermogenesis

In the format provided by the authors and unedited

SUPPLEMENTARY INFORMATION

SUPPLEMENTARY TABLE 1 ***PARTICIPANT CHARACTERISTICS FOR ADIPOSE TISSUE COLLECTIONS.***

Experiment/ Subject number	Operation	Underlying diagnosis	Age (years)	Sex	BMI (Kg/m^2)
**Whole tissue mRNA and histology**					
**1**	Parathyroidectomy	Primary hyperparathyroidism	66	Female	28.3
**2**	Parathyroidectomy	Primary hyperparathyroidism	63	Female	33.3
**3**	Thyroid lobectomy	Papillary carcinoma of thyroid	45	Female	26.5
**4**	Thyroid lobectomy	Follicular adenoma	48	Female	17.8
**5**	Parathyroidectomy	Primary hyperparathyroidism	47	Male	23.4
**6**	Parathyroidectomy	Primary hyperparathyroidism	24	Male	27.7
**7**	Thyroidectomy	Graves' disease	27	Female	30.1
**8**	Thyroidectomy	Graves' disease	26	Female	20.9
**9**	Parathyroidectomy	Primary hyperparathyroidism	62	Female	28.4
**10**	Parathyroidectomy	Primary hyperparathyroidism	56	Male	27.2
**Cultured adipocytes for RNA seq**					
**1**	Thyroid lobectomy	Follicular thyroid carcinoma	41	Female	27.5
**2**	Parathyroidectomy	Primary hyperparathyroidism	59	Female	24.5
**3**	Thyroidectomy	Follicular thyroid carcinoma	52	Female	39.4
**4**	Thyroidectomy	Multinodular goitre	50	Female	22.3
**Adipocytes for qPCR**					
**1**	Parathyroidectomy	Primary hyperparathyroidism	51	Female	26.3
**2**	Thyroid lobectomy	Benign hyperplastic nodule	41	Female	28.6
**3**	Thyroidectomy	Follicular carcinoma	35	Female	37.0
**4**	Parathyroidectomy	Primary hyperparathyroidism	70	Female	32.5
**5**	Parathyroidectomy	Primary hyperparathyroidism	58	Female	22.9
**6**	Parathyroidectomy	Primary hyperparathyroidism	59	Female	24.4
**7**	Thyroid lobectomy	Papillary thyroid carcinoma	36	Female	28.0
**8**	Parathyroidectomy	Primary hyperparathyroidism	61	Female	23.3
**9**	Parathyroidectomy	Primary hyperparathyroidism	67	Female	18.7
**10**	Parathyroidectomy	Primary hyperparathyroidism	62	Female	30.8
**11**	Parathyroidectomy	Primary hyperparathyroidism	63	Female	33.9
**12**	Parathyroidectomy	Primary hyperparathyroidism	68	Female	21.9
**Serotonin uptake**					
**1**	Parathyroidectomy	Primary hyperparathyroidism	45	Female	40.8
**2**	Parathyroidectomy	Primary hyperparathyroidism	60	Female	24.7
**3**	Parathyroidectomy	Primary hyperparathyroidism	51	Female	26.3
**4**	Parathyroidectomy	Primary hyperparathyroidism	59	Male	39.4
**5**	Thyroid lobectomy	Multinodular goitre	41	Female	28.6
**6**	Parathyroidectomy	Primary hyperparathyroidism	61	Female	22.6
**Serotonin Respirometry**					
**1**	Parathyroidectomy	Primary hyperparathyroidism	74	Female	21.0
**2**	Parathyroidectomy	Primary hyperparathyroidism	58	Female	22.9
**3**	Parathyroidectomy	Primary hyperparathyroidism	59	Female	24.4

**4**	Parathyroidectomy	Primary hyperparathyroidism	49	Female	36.0
**5**	Parathyroidectomy	Primary hyperparathyroidism	47	Female	37.6
**6**	Thyroid Lobectomy	Papillary carcinoma of thyroid	36	Female	28.0
**7**	Parathyroidectomy	Primary hyperparathyroidism	69	Female	30.5
**8**	Parathyroidectomy	Primary hyperparathyroidism	62	Female	30.8
**9**	Thyroid lobectomy	Follicular adenoma	29	Female	21.2
**10**	Parathyroidectomy	Primary hyperparathyroidism	62	Female	22.4
**11**	Thyroidectomy	Graves' disease	68	Female	28.3
**12**	Thyroid lobectomy	Hurthle cell carcinoma	61	Female	30.5
**13**	Parathyroidectomy	Primary hyperparathyroidism	76	Male	26.5
**14**	Parathyroidectomy	Primary hyperparathyroidism	63	Female	27.1
**Serotonin regulation of UCP1**					
**1**	Parathyroidectomy	Primary hyperparathyroidism	21	Female	30.1
**2**	Parathyroidectomy	Primary hyperparathyroidism	52	Female	35.7
**3**	Parathyroidectomy	Primary hyperparathyroidism	36	Male	37.0
**4**	Parathyroidectomy	Primary hyperparathyroidism	56	Female	31.2
**5**	Parathyroidectomy	Primary hyperparathyroidism	70	Female	27.9
**6**	Parathyroidectomy	Primary hyperparathyroidism	58	Female	23.8
**7**	Parathyroidectomy	Primary hyperparathyroidism	70	Female	32.5
**8**	Parathyroidectomy	Primary hyperparathyroidism	74	Female	21.0
**9**	Parathyroidectomy	Primary hyperparathyroidism	57	Female	49.5
**10**	Parathyroidectomy	Primary hyperparathyroidism	58	Female	22.9
**11**	Parathyroidectomy	Primary hyperparathyroidism	59	Female	24.4
**12**	Parathyroidectomy	Primary hyperparathyroidism	49	Female	36.0
**13**	Parathyroidectomy	Primary hyperparathyroidism	55	Female	27.5
**14**	Parathyroidectomy	Primary hyperparathyroidism	47	Female	37.6
**Serotonin/ SSRI respirometry**					
**1**	Thyroid lobectomy	Hurthle cell carcinoma	54	Female	23.7
**2**	Parathyroidectomy	Primary hyperparathyroidism	76	Male	26.5
**3**	Parathyroidectomy	Primary hyperparathyroidism	63	Female	27.1
**4**	Parathyroidectomy	Primary hyperparathyroidism	36	Female	19.7
**5**	Graves' disease	Thyroidectomy	51	Female	23.2
**6**	Parathyroidectomy	Primary hyperparathyroidism	40	Male	26.6
**7**	Parathyroidectomy	Primary hyperparathyroidism	26	Male	31.4
**8**	Graves' disease	Thyroidectomy	65	Male	19.8
**9**	Multinodular goitre	Thyroidectomy	32	Female	24.9
**Serotonin/ SSRI regulation of UCP1**					
**1**	Thyroid lobectomy	Papillary carcinoma of thyroid	36	Female	28.0
**2**	Parathyroidectomy	Primary hyperparathyroidism	69	Female	30.5
**3**	Parathyroidectomy	Primary hyperparathyroidism	67	Female	18.7
**4**	Parathyroidectomy	Primary hyperparathyroidism	62	Female	30.8
**5**	Parathyroidectomy	Primary hyperparathyroidism	68	Female	21.9
**6**	Thyroidectomy	Graves' disease	68	Female	28.3
**7**	Parathyroidectomy	Primary hyperparathyroidism	76	Male	26.5
**8**	Parathyroidectomy	Primary hyperparathyroidism	31	Female	32.1
**9**	Parathyroidectomy	Primary hyperparathyroidism	71	Female	29.3

**5-HT receptor regulation of UCP1**					
**1**	Parathyroidectomy	Primary hyperparathyroidism	70	Female	28.5
**2**	Parathyroidectomy	Primary hyperparathyroidism	68	Female	29.4
**3**	Parathyroidectomy	Primary hyperparathyroidism	60	Female	31.5
**4**	Parathyroidectomy	Primary hyperparathyroidism	36	Female	50.3
**5**	Thyroidectomy	Graves' disease	53	Female	27.3
**6**	Thyroidectomy	Hurthle cell adenomas	47	Female	19.8
**7**	Thyroidectomy	Graves' disease	38	Female	27.5
**8**	Thyroidectomy	Graves' disease	65	Male	23.5
**9**	Parathyroidectomy	Primary hyperparathyroidism	51	Male	28.0
**10**	Thyroid Lobectomy	Multinodular goitre	64	Female	30.8
**HTR2A/B knockdown**					
**1**	Thyroidectomy	Follicular adenoma	67	Male	33.7
**2**	Parathyroidectomy	Primary hyperparathyroidism	49	Female	22.9
**3**	Graves' disease	Thyroidectomy	65	Male	19.8
**4**	Parathyroidectomy	Primary hyperparathyroidism	73	Female	24.0
**5**	Multinodular goitre	Thyroidectomy	32	Female	24.9
**6**	Parathyroidectomy	Primary hyperparathyroidism	59	Female	29.8
