# Supplementary material for: The serotonin transporter sustains human brown adipose tissue thermogenesis
Source: Nat Metab. 2023 Aug 3;5(8):1319–36. doi: 10.1038/s42255-023-00839-2 (PMC10447248; doi:10.1038/s42255-023-00839-2)
Supplement: Supplementary file 2 — Reporting Summary [file 42255_2023_839_MOESM2_ESM.pdf]

nature portfolio

Corresponding author(s): Roland Stimson

Last updated by author(s): May 29, 2023

Reporting Summary

Nature Portfolio wishes to improve the reproducibility of the work that we publish. This form provides structure for consistency and transparency in reporting. For further information on Nature Portfolio policies, see our Editorial Policies and the Editorial Policy Checklist.

Statistics

For all statistical analyses, confirm that the following items are present in the figure legend, table legend, main text, or Methods section.

n/a	Confirmed	
☐	☒	The exact sample size (*n*) for each experimental group/condition, given as a discrete number and unit of measurement
☐	☒	A statement on whether measurements were taken from distinct samples or whether the same sample was measured repeatedly
☐	☒	The statistical test(s) used AND whether they are one- or two-sided<br>*Only common tests should be described solely by name; describe more complex techniques in the Methods section.*
☐	☒	A description of all covariates tested
☐	☒	A description of any assumptions or corrections, such as tests of normality and adjustment for multiple comparisons
☐	☒	A full description of the statistical parameters including central tendency (e.g. means) or other basic estimates (e.g. regression coefficient) AND variation (e.g. standard deviation) or associated estimates of uncertainty (e.g. confidence intervals)
☐	☒	For null hypothesis testing, the test statistic (e.g. *F*, *t*, *r*) with confidence intervals, effect sizes, degrees of freedom and *P* value noted<br>*Give P values as exact values whenever suitable.*
☒	☐	For Bayesian analysis, information on the choice of priors and Markov chain Monte Carlo settings
☒	☐	For hierarchical and complex designs, identification of the appropriate level for tests and full reporting of outcomes
☐	☒	Estimates of effect sizes (e.g. Cohen's *d*, Pearson's *r*), indicating how they were calculated

Our web collection on statistics for biologists contains articles on many of the points above.

Software and code

Policy information about availability of computer code

Data collection	No software was used.
Data analysis	RNA sequencing. Gene expression level normalisation was performed using Bioconductor version 3.10, DESeq2_1.26.0, which was used for downstream analysis. Pathway analysis of gene identifiers extracted from RNA-seq was performed using Ingenuity Pathway Analysis (IPA, Ingenuity® systems, www.ingenuity.com, Qiagen). Prism software version 9 (GraphPad, USA) and SPSS version 25 (IBM) were used for statistical analyses.

For manuscripts utilizing custom algorithms or software that are central to the research but not yet described in published literature, software must be made available to editors and reviewers. We strongly encourage code deposition in a community repository (e.g. GitHub). See the Nature Portfolio guidelines for submitting code & software for further information.

Data

Policy information about availability of data

All manuscripts must include a data availability statement. This statement should provide the following information, where applicable:

- Accession codes, unique identifiers, or web links for publicly available datasets
- A description of any restrictions on data availability
- For clinical datasets or third party data, please ensure that the statement adheres to our policy

All data generated or analysed during this study are included in this published article (including its supplementary information files). The RNA sequencing data generated during this study are available on ArrayExpress (E-MAB-101123).

Research involving human participants, their data, or biological material

Policy information about studies with human participants or human data. See also policy information about sex, gender (identity/presentation), and sexual orientation and race, ethnicity and racism.

Reporting on sex and gender	We have reported the sex of the subjects in all of the human studies, including the cellular experiments. Both sexes were purposefully recruited to in vivo healthy volunteer studies, in the sertraline study all subjects acted as their own control and in the case-control study a similar proportion of females/ males were recruited to avoid bias. In the retrospective PET/CT analysis, all 3 groups were sex-matched. In the two healthy volunteer studies we show the individual data points and have used different colours for the female and males to make it easier for the reader. We report a similar effect of sertraline independent of sex in these volunteers and report on the sex difference in the retrospective PET/CT analysis.
Reporting on race, ethnicity, or other socially relevant groupings	We have not reported on ethnicity in this manuscript, for the PET/CT retrospective analysis that data was not available. For the healthy volunteer studies, the numbers recruited were too small to allow any subgroup analysis of ethnicity and in the sertraline healthy volunteer study all subjects acted as their own control.
Population characteristics	1. In vivo crossover study in healthy volunteers - 15 healthy volunteers (9 female, 6 male) aged 18-35y . Inclusion criteria: body mass index (BMI) 18.5–25 Kg/m2; weight change of <5% in preceding 6 months; no acute or chronic medical conditions; on no regular medications; alcohol intake ≤14 units per week; no claustrophobia or contraindication to MRI scanning; no pregnancy or breastfeeding in female subjects; normal screening blood tests (full blood count, glucose, kidney, liver, and thyroid function).<br><br>2. In vivo study in normal weight and obese healthy volunteers - 20 healthy volunteers aged 18-40y (Table 2) were recruited to a case control study . Inclusion criteria: BMI 18.5–25 Kg/m2 (normal weight group; 5 male and 5 female) or 30-55 Kg/m2 (obese group; 4 male and 6 female); weight change of <5% in preceding 6 months; no acute or chronic medical conditions known to alter brown adipose tissue activity or preclude an abdominal adipose tissue biopsy; on no regular medications known to alter serotonin concentrations or BAT activity; alcohol intake ≤14 units per week; no pregnancy or breastfeeding in female subjects; no allergy to local anaesthetic; normal screening blood tests (full blood count, glucose, kidney, liver, and thyroid function).<br><br>3. Retrospective analysis of patient PET/CT scans. The records of patients who had undergone 18F-FDG-PET/CT scanning between December 2014 and January 2016 or from November 2017 to June 2019. Subjects were identified who were currently prescribed a selective serotonin re-uptake inhibitor (SSRI) (n=153 patients identified) and 2 matched control groups were also identified, those currently prescribed any other class of anti-depressant (n=164) and those not currently prescribed an antidepressant medication (n=270). Participants were matched for sex, age, body weight, fasting glucose, presence of diabetes, underlying cancer diagnosis and outdoor temperature in the month of scan.<br><br>We report at least the mean age, sex, weight and body mass index of all the human volunteer recruited to these studies, including individual data for the patients used for all the individual cellular experiments.
Recruitment	Participants to the two healthy volunteer studies were identified either by advert (newspaper, poster or email) or by using the SHARE resource. Bias was minimised by having strict eligibility criteria for these subjects, younger adult subjects were recruited in order to maximise the number of healthy volunteers with detectable brown adipose tissue so these findings may not be relevant to older subjects. Patients due to undergo elective neck surgery were identified for recruitment to the cellular studies. Only patients with normal thyroid function were recruited to these studies to minimise confounders.
Ethics oversight	1. Approval was obtained from the South East Scotland Research Ethics Committee (ethics number 18/SS/0104) and informed consent was obtained from each subject.<br>2. Approval was obtained from the Edinburgh Medical School Research Ethics Committee (ethics number 18-HV-049) and informed consent was obtained from each subject.<br>3. Local Caldicott guardian approval was obtained<br>Approval for the human tissue collections was obtained from the East of Scotland Research Ethics Committee (ethics numbers 15/ES/0094 and 20/ES/0061).

Note that full information on the approval of the study protocol must also be provided in the manuscript.

Field-specific reporting

Please select the one below that is the best fit for your research. If you are not sure, read the appropriate sections before making your selection.

☒ Life sciences ☐ Behavioural & social sciences ☐ Ecological, evolutionary & environmental sciences

For a reference copy of the document with all sections, see nature.com/documents/nr-reporting-summary-flat.pdf

Life sciences study design

All studies must disclose on these points even when the disclosure is negative.

Sample size	Power analysis was conducted to determine sample size.
Data exclusions	No data were excluded.
Replication	Statistical testing was only performed using independent biological replicates rather than technical replicates. The numbers presented in the manuscript for each experiment represent independent biological samples that were used to ensure there was reproducibility of the experimental findings.
Randomization	Randomization of the sertraline healthy volunteer study was performed by an unrelated party (NHS Scotland Pharmaceutical 'Specials' Service).
Blinding	The healthy volunteer sertraline study was a double-blind randomised crossover study and investigators remained blinded until completion of all the analyses. Volunteers could not be blinded to their respective groups in the case-control study of normal weight and obese healthy volunteers, however investigators undertaking the analyses were blinded to the groups until completion of the analyses.

Reporting for specific materials, systems and methods

We require information from authors about some types of materials, experimental systems and methods used in many studies. Here, indicate whether each material, system or method listed is relevant to your study. If you are not sure if a list item applies to your research, read the appropriate section before selecting a response.

Materials & experimental systems

n/a	Involved in the study	
☐	☒	Antibodies
☒	☐	Eukaryotic cell lines
☒	☐	Palaeontology and archaeology
☐	☒	Animals and other organisms
☐	☒	Clinical data
☒	☐	Dual use research of concern
☒	☐	Plants

Methods

n/a	Involved in the study	
☒	☐	ChIP-seq
☒	☐	Flow cytometry
☒	☐	MRI-based neuroimaging

Antibodies

Antibodies used	Primary antibodies: 1) mouse anti-human SERT monoclonal antibody (1:1500 dilution, Millipore, Feltham, UK, IgG, Catalog number MAB5618, LOT 2964467); 2) rabbit anti-human UCP1 polyclonal antibody (1:8000 dilution, Sigma-Aldrich, Merck Life Science, Gillingham, UK, Catalog number U6382, LOT 080M4767). Secondary antibodies: 1) Goat anti-mouse IgG polyclonal antibody (1:800 dilution, Vector laboratories, CA, USA, Catalog number BA-9200, LOT ZB0324); 2) Goat anti-rabbit IgG polyclonal antibody (1:800 dilution, Vector laboratories, CA, USA, Catalog number BA-1000, LOT VO527).
Validation	We have published the anti-UCP1 antibody previously (Ramage et al, Cell Metabolism 2016; 24(1): 130-41) and has been validated in other publications including Chondronikola M et al, Diabetes 2014; 63(12): 4089–4099). The anti-SERT antibody has been validated previously in several publications including Alkemade A et al, Brain structure & function 2019; 224(9): 3213-3227 and Borgers AJ et al, Frontiers in Neuroscience 2014; 8: 106.

Animals and other research organisms

Policy information about studies involving animals; ARRIVE guidelines recommended for reporting animal research, and Sex and Gender in Research

Laboratory animals	Murine inguinal, epididymal and brown adipose tissue was collected from 8-9-week old male 129/Ola mice. Mice were housed at 20-21°C and at a humidity of 50-55%, using a 12/12h light/dark cycle.

Wild animals	No wild animals were used.
Reporting on sex	Tissues were collected from male mice. qPCR was performed in the whole tissue of 4 mice and in cultured adipocytes from another 4 mice.
Field-collected samples	No field collected samples were used.
Ethics oversight	No ethical approval was required (schedule 1 wild-type mice) as these mice were not part of a specific experiment and were surplus to requirements, as such no experimental research form was required. Tissues were collected immediately post-procedure .

Note that full information on the approval of the study protocol must also be provided in the manuscript.

Clinical data

Policy information about clinical studies

All manuscripts should comply with the ICMJE guidelines for publication of clinical research and a completed CONSORT checklist must be included with all submissions.

Clinical trial registration	Not applicable, none of the human studies were classed as clinical trials
Study protocol	Protocol for sertraline crossover study provided.
Data collection	Described above.
Outcomes	The change in 18F-fluorodeoxyglucose uptake by BAT between sertraline and placebo phases was the primary outcome measure for the sertraline crossover study. The difference in circulating serotonin concentrations between normal weight and obese volunteers was the primary outcome measure for the case-control study. The difference between groups relating to the number of patients with detectable 18F-fluorodeoxyglucose uptake by BAT was the main outcome measure for the retrospective PET/CT analysis.
